# miRNA-seq identification and clinical validation of CD138+ and circulating miR-25 in treatment response of multiple myeloma

**DOI:** 10.1186/s12967-023-04034-5

**Published:** 2023-04-06

**Authors:** Maria-Alexandra Papadimitriou, Konstantinos Soureas, Aristea-Maria Papanota, Panagiotis Tsiakanikas, Panagiotis G. Adamopoulos, Ioannis Ntanasis-Stathopoulos, Panagiotis Malandrakis, Maria Gavriatopoulou, Diamantis C. Sideris, Efstathios Kastritis, Margaritis Avgeris, Meletios-Athanasios Dimopoulos, Evangelos Terpos, Andreas Scorilas

**Affiliations:** 1 Department of Biochemistry and Molecular Biology, Faculty of Biology, National and Kapodistrian University of Athens, Panepistimiopolis, 15771 Athens, Greece; 2 Laboratory of Clinical Biochemistry–Molecular Diagnostics, Second Department of Pediatrics, School of Medicine, National and Kapodistrian University of Athens, “P. & A. Kyriakou” Children’s Hospital, Athens, Greece; 3 Department of Clinical Therapeutics, School of Medicine, National and Kapodistrian University of Athens, “Alexandra” General Hospital, 80 Vas. Sofias Ave., 11528 Athens, Greece

**Keywords:** miRNA, miRNA-seq, Small RNA-seq, CD138 + plasma cells, Hematological malignancies, Non-coding RNAs

## Abstract

**Background:**

Despite significant advancements in multiple myeloma (MM) therapy, the highly heterogenous treatment response hinders reliable prognosis and tailored therapeutics. Herein, we have studied the clinical utility of miRNAs in ameliorating patients’ management.

**Methods:**

miRNA-seq was performed in bone marrow CD138+ plasma cells (PCs) of 24 MM and smoldering MM (sMM) patients to analyze miRNAs profile. CD138+ and circulating miR-25 levels were quantified using *in house* RT-qPCR assays in our screening MM/sMM cohort (CD138+ plasma cells n = 167; subcohort of MM peripheral plasma samples n = 69). Two external datasets (Kryukov et al*.* cohort n = 149; MMRF CoMMpass study n = 760) served as institutional-independent validation cohorts. Patients’ mortality and disease progression were assessed as clinical endpoints. Internal validation was performed by bootstrap analysis. Clinical benefit was estimated by decision curve analysis.

**Results:**

miRNA-seq highlighted miR-25 of CD138+ plasma cells to be upregulated in MM *vs.* sMM, R-ISS II/III *vs.* R-ISS I, and in progressed compared to progression-free patients. The analysis of our screening cohort highlighted that CD138+ miR-25 levels were correlated with short-term progression (HR = 2.729; p = 0.009) and poor survival (HR = 4.581; p = 0.004) of the patients; which was confirmed by Kryukov et al*.* cohort (HR = 1.878; p = 0.005) and MMRF CoMMpass study (HR = 1.414; p = 0.039) validation cohorts. Moreover, multivariate miR-25-fitted models contributed to superior risk-stratification and clinical benefit in MM prognostication. Finally, elevated miR-25 circulating levels were correlated with poor survival of MM patients (HR = 5.435; p = 0.021), serving as a potent non-invasive molecular prognostic tool.

**Conclusions:**

Our study identified miR-25 overexpression as a powerful independent predictor of poor treatment outcome and post-treatment progression, aiding towards modern non-invasive disease prognosis and personalized treatment decisions.

**Supplementary Information:**

The online version contains supplementary material available at 10.1186/s12967-023-04034-5.

## Background

Multiple myeloma (MM) represents the second most prevalent hematologic malignancy, accounting for approximately 20% of blood cancer-related mortality in developed countries [1, 2]. MM is generally preceded by the benign monoclonal gammopathy of undetermined significance (MGUS), which can later progress through an asymptomatic smoldering (sMM) phase before manifesting clinical symptoms warranting therapy [3, 4]. The transition among disease stages and treatment resistance is correlated with complex pathophysiology, in which DNA damage, genomic instability, epigenetic defects and the bone marrow (BM) microenvironment components lead to the development and progression of myeloma cells [5–7].

Despite the significantly improved response rates and survival of MM patients, since the development of novel anti-MM drugs in the past two decades, MM remains an incurable malignancy due to the emergence of relapse and treatment resistance in its clinical course [8, 9]. Nowadays, a uniform approach to determine MM prognosis remains elusive despite several validated risk-stratification systems applied in clinical practice, such as the Revised International Staging System (R-ISS) [10, 11]. Most importantly, MM monitoring relies on lifelong surveillance with invasive interventions, mainly BM biopsies, which however are subjected to significant intra- and inter-observational variability, adversely affecting patients’ quality-of-life and leading to increased economic burden for healthcare systems [12]. In this regard, the identification of predictive molecular markers supporting personalized prognosis and tailored therapeutics is of utmost importance to improve patients’ survival and disease management.

MicroRNAs (miRNAs; 18–25 nt in length), have been unveiled as essential part of the non-coding transcriptome, holding a pivotal role in post-transcriptional regulation of gene expression [13, 14]. The miRNA regulatory function is exerted mainly through binding to the 3′-untranslated region (3′-UTR) of their target mRNAs and recruiting the RNA-induced silencing complex -RISC-, which in turn mediates the inhibition of translation and mRNA decay. A great number of miRNAs display high conservation in their “seed region” across species, while more than 60% of protein-coding genes are validated to be targeted by multiple miRNAs, unveiling the complex and profound effects of miRNAs on physiological and developmental processes [15, 16]. In this regard, miRNAs have arisen as key mediators of a wide range of biological procedures, such as cell proliferation, apoptosis and migration, while their aberrant expression has been associated with numerous human malignancies including MM, with comprehensive evidence supporting their potent prognostic value [17–20].

In recent years, critical advancements in high-throughput technologies have enabled the global and accurate identification of differentially expressed miRNAs and the construction of miRNA prognostic signatures. The aim of our present study is to provide insight into the miRNA landscape in MM aiming to reveal potential MM-related miRNAs of prognostic significance for patients’ treatment. Using miRNA-seq of CD138+ plasma cells, miR-25 was highlighted to be overexpressed in MM patients with short-term disease progression, and thereafter, the clinical value of miR-25 in MM prognosis and treatment outcome was further evaluated, for the first time, in a screening MM/sMM cohort (n = 167 patients) and two external institutional-independent validation cohorts, the Multiple Myeloma Research Foundation (MMRF) CoMMpass study (MMRF CoMMpass n = 760) and the Kryukov et al*.* cohort (n = 149) [21]. Our study highlighted that CD138+ overexpression of miR-25 was strongly associated with worse post-treatment survival, and contributed to superior clinical benefit in MM prognostication. Prompted by the independent clinical value of CD138+ miR-25 overexpression, we further evaluated its significance in patients’ pre-treatment peripheral plasma. Strikingly, the unfavorable prognostic utility of miR-25 overexpression was maintained in patients’ circulation, supporting modern non-invasive disease prognosis and management.

## Methods

### Screening cohort

The screening cohort of the study consisted of CD138+ plasma cells from 167 patients diagnosed with MM (n = 139) and sMM (n = 28), while for a subset cohort of 69 MM patients, peripheral blood plasma samples were also available. BM aspirates and whole blood samples were collected during disease diagnosis at the Department of Clinical Therapeutics, “Alexandra” Hospital, Athens, Greece. All MM patients included in the study were newly diagnosed, non-previously administered treatment, and their diagnosis was based on the standard criteria of the International Myeloma Working Group (IMWG) [22]. At the time of diagnosis, patients were subjected to baseline assessment using blood, biochemical and imaging tests. Cytogenetic abnormalities were identified using conventional cytogenetic protocols as well as fluorescence in situ hybridization (FISH) at BM aspirate from trephine biopsy. Bone disease was assessed by whole-body low dose computed tomography. Focusing on MM patients, 24.5%, 48.9% and 20.1% of the patients were classified as R-ISS I, II and III, respectively; 81 patients presented  ≥ 60% BM infiltration by plasma cells, while bone disease was detected in 66.2% of the patients at diagnosis.

Of the 139 recruited patients, 122 (87.8%) patients received bortezomib-based regimens, while 12 (8.6%) of them were treated with lenalidomide-based regimens. High dose melphalan with autologous stem cell transplantation (HDM/ASCT) was administrated to 38 patients. Response to treatment was assessed monthly according to IMWG criteria with blood and urine tests [23]. Follow-up was successfully completed for 135 patients (97.1%). Due to unclear monitoring data, 4 patients (2.9%) were excluded from the survival analysis. The median follow-up was 24 months (95% CI 22.49–25.51), in which death and disease relapse were detected in 30 (22.2%) and 33 (24.4%) MM patients, respectively. Regarding patients’ clinical outcome, the progression-free survival (PFS) was 23.7 (95% CI 21.79–25.58) months, while patients displayed a mean overall survival (OS) of 27.2 months (95% CI 25.59–28.73).

**Fig. 1:**
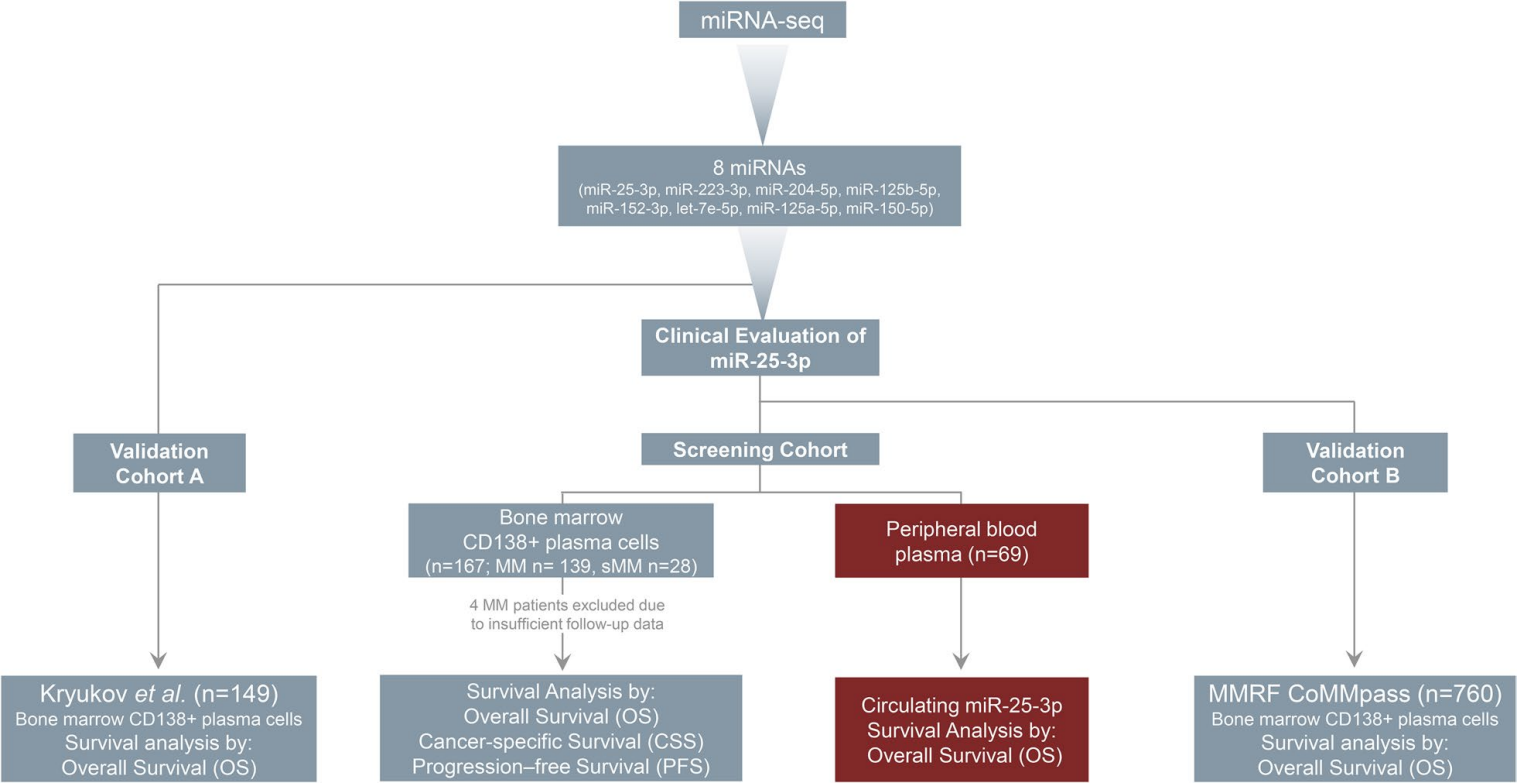
REMARK diagram of the study

The study was performed with respect to ethical standards of the 1975 Declaration of Helsinki, as revised in 2008, and approved by the Ethics Committee of “Alexandra” Hospital, Athens, Greece. Prior to sampling, informed consent was acquired from all participating patients. The study’s REMARK diagram is presented in Fig. 1, whereas patients’ clinicopathological data are summarized in Table 1.

**Table 1:** Clinicopathological features of the screening cohort

Variable	No. of patients n = 167	
	Bone marrow CD138+ plasma cells n = 167	Peripheral blood plasma n = 69
Disease		
sMM	28 (16.8%)	–
MM	139 (83.2%)	69 (100.0%)
Multiple myeloma patients		
R-ISS stage		
R-ISS I	34 (24.5%)	20 (29.0%)
R-ISS II	68 (48.9%)	28 (40.6%)
R-ISS III	28 (20.1%)	14 (20.3%)
No data	9	7
ISS Stage		
ISS I	40 (28.8%)	25 (36.2%)
ISS II	43 (30.9%)	19 (27.5%)
ISS III	55 (39.6%)	24 (34.8%)
No data	1	1
Gender		
Male	77 (55.4%)	38 (55.1%)
Female	62 (44.6%)	31 (44.9%)
Therapy		
Bortezomib-based regimens	122 (87.8%)	65 (94.2%)
Lenalidomide-dexamethasone	12 (8.6%)	4 (5.8%)
Other	2 (1.4%)	0
Not complete treatment	3 (2.2%)	0
Bone disease		
Yes	92 (66.2%)	42 (60.9%)
No	38 (27.3%)	27 (39.1%)
No data	9	0
HDM/ASCT		
Yes	38 (27.3%)	28 (40.6%)
No	100 (71.9%)	41 (59.4%)
No data	1	0
B2M		
< 3.5 mg/l	43 (30.9%)	26 (37.7%)
≥ 3.5 mg/l and < 5.5 mg/l	41 (29.5%)	19 (27.5%)
> 5.5 mg/l	54 (38.8%)	23 (33.3%)
No data	1	1
LDH		
≤ 220 U/l	107 (77.0%)	54 (78.3%)
> 220 U/l	32 (23.0%)	15 (21.7%)
Marrow plasma cells		
< 60%	58 (41.7%)	33 (47.8%)
≥ 60%	81 (58.3%)	36 (52.2%)
Response to 1st line		
sCR / CR / VGPR	90 (64.7%)	47 (68.1%)
PR / SD / PD	45 (32.4%)	22 (30.4%)
No data	4	0
Disease monitoring		
Follow-up patients	135	69
Relapse	33 (24.4%)	17 (24.6%)
Death	30 (22.2%)	13 (18.8%)
Progression	49 (36.3%)	25 (36.2%)
Excluded from follow-up	4	0

HDM/ASCT: high-dose melphalan therapy with autologous stem cell transplantation, sCR: stringent complete response, CR: complete response, VGPR: very good partial response, PR: partial response, SD: stable disease, PD: progressed disease

### Validation cohorts

The MMRF CoMMpass study (n = 787; NCT01454297) and the Kryukov et al*.* cohort (n = 151) [21] were used as independent validation cohorts. The MMRF CoMMpass study performed RNA-seq in CD138+ plasma cells from 787 patients with primary MM; while complete follow-up data are available for 760 patients. Kryukov et al*.* cohort consists of 151 MM patients and transcriptional profiling of CD138+ plasma cells was generated by microarray analysis with the Affymetrix GeneChip Human Gene 1.0 ST Array platform; while complete follow-up data are available for 149 patients. Clinical and normalized sequencing data were downloaded for MMRF CoMMpass by Genomic Data Commons (GDC) data portal (https://portal.gdc.cancer.gov/) and for the Kryukov et al*.* dataset by EMBL-EBI ArrayExpress (E-MTAB-1038 and E-MTAB-4032).

### CD138+ plasma cells and blood plasma isolation

BM aspirates were collected in EDTA tubes and Ficoll-Paque was used for the isolation of mononuclear cells. CD138+ plasma cells were enriched by magnetic cell sorting with immunomagnetic microbeads coated with anti-CD138 monoclonal antibody (MACS CD138 microbeads, Miltenyi-Biotec GmbH, Bergisch Gladbach, Germany).

Peripheral whole blood samples were available from 69 patients of the screening cohort and collected in EDTA tubes. Sequential centrifuges (1500 g for 10 min twice, and 17000 g for 10 min) were applied to remove the blood cells and the cellular debris, and the extracted peripheral blood plasma was stored at − 80 °C until analysis. Free hemoglobin levels were evaluated spectrophotometrically using the Harboe method and Allen correction and hemolyzed plasma samples were excluded from the analysis.

### RNA extraction

After plasma cells purification, total RNA from CD138+ plasma cells was extracted using TRI-Reagent (Molecular Research Center, Cincinnati, OH, USA) in line with the manufacturer’s protocol. RNA was eluted in RNA Storage Solution (RSS; Ambion, Austin, TX, USA) while its concentration was determined using the Qubit RNA Broad Range assay in the Qubit 2.0 Fluorometer (Invitrogen, Carlsbad, CA, USA).

Circulating miRNAs were isolated from 300 μL of fresh frozen plasma samples using NucleoSpin miRNA Plasma kit (Macherey–Nagel GmbH & Co. KG, Duren, Germany) according to manufacturer’s instructions and stored at − 80 °C until further analysis.

### miRNA-seq

Next Generation Sequencing (NGS) libraries were constructed from 4 R-ISS I, 8 R-ISS II, 8 R-ISS III MM and 4 sMM patients, using the QIAseq miRNA Library Kit (Qiagen, Hilden, Germany) and 500 ng total RNA of CD138+ plasma cells as starting template. Briefly, adapters were ligated to the 3'- and 5'-ends of miRNAs sequentially, UMI (Unique Molecular Identifier)-based cDNA synthesis and cleanup was performed, followed by amplification with a universal forward primer and indexing reverse primers. The quality assessment of the constructed libraries was carried out in the 2100 Bioanalyzer (Agilent Technologies, Santa Clara, CA, USA), using the Agilent High Sensitivity DNA Kit (Agilent). Finally, the miRNA-seq was performed using Illumina NextSeq 550 system (Illumina, San Diego, CA, USA), producing 75 bp single sequencing reads for each miRNA-seq library.

### Bioinformatic analysis

The quality control of the sequenced libraries was accomplished with FastQC software, while trim galore algorithm performed adapter trimming. Sequencing reads with lengths  < 16 nt and  > 40 nt were excluded from the datasets used for downstream analysis. Subsequently, miRDeep2 was used for mapping mature miRNAs [24] (according miRbase database release 22.1 [25]) on human genome sequence (hg38) and miRNA expression profiling across the investigated samples. For each tested sample, only miRNA candidates with positive miRDeep2 score and > 50 unnormalized read counts were used for further analysis.

### Gene ontology (GO) enrichment analysis

miRDB [26], TargetScanHuman 8.0 [27] and DIANA TOOLS—microT-CDS [28] target prediction tools were used for the prediction of the potential miRNA target genes. To minimize the prediction error rate, specific inclusion criteria were applied and only targets with miRDB Target Score ≥ 80, TargetScan cumulative weighted context++ score ≤ − 0.3 and DIANA micro-T-CDS prediction score ≥ 0.8 were selected. Target genes were analyzed for Gene ontology (GO) enrichment through Enrichr database platform [29]. Biological processes (BPs), cellular components (CCs), and molecular functions (MFs) with a q-value (FDR adjusted p-value) < 0.05, and combined enrichment score > 10 were retained. The retrieved GO terms were imported to REVIGO [30] where they were clustered based on their similarity and any potential redundancy was removed, while GOnet [31] was used to visualize relationships between miR-25 target genes and statistically significant GO BP terms.

### Polyadenylation of total RNA and first-strand cDNA synthesis

Total RNA was polyadenylated at 3-end in a 10 µL reaction, containing 200 ng of total RNA from CD138+ plasma cells or 2 µL total RNA from plasma samples, 1 U of recombinant *E.coli* Poly(A) Polymerase (New England Biolabs Inc., Ipswich, MA, USA) and 800 µM ATP, at 37 ºC for 60 min. Subsequently, the enzyme was inactivated at 65 °C for 10 min.

Following polyadenylation, reverse transcription of the RNA was performed in a 20 μL reaction containing 200 U M-MLV Reverse Transcriptase (Invitrogen), 40 U RNaseOUT Recombinant Ribonuclease Inhibitor (Invitrogen), 10 mM dNTP Mix and 250 mM oligo-dT adapter 5′-GCGAGCACAGAATTAATACGACTCACTATAGGTTTTTTTTTTTTVN-3′ (V = G, A, C and N = G, A, T, C), at 37 ºC for 60 min. Thereafter, heat inactivation of the enzyme was accomplished at 70 °C for 15 min.

### Quantitative real-time PCR (qPCR)

Quantitative real-time PCR (qPCR) assays using SYBR Green I dye were developed and applied in a total of 236 samples (CD138+ plasma cells n = 167; peripheral plasma samples n = 69), for the quantification of miR-25 levels. Based on published sequences (NCBI RefSeq: NR_002745.1 for SNORD48, NR_029486 for miR-16-5p and NR_029498.1 for miR‐25-3p), specific forward primers for miR-25-3p (5′‐ ATTGCACTTGTCTCGGTCTGA‐3′), small nucleolar RNA, C/D box 48 (SNORD48), frequently annotated as RNU48, (5′-TGATGATGACCCCAGGTAACTCT-3′) and miR-16-5p (5′-TAGCAGCACGTAAATATTGGCG‐3′) were synthesized. The specific forward primers and a universal reverse primer (5′-GCGAGCACAGAATTAATACGAC-3′), complementary to the above-mentioned oligo-dT adapter, were used for the amplification of a 62 bp specific amplicon of miR-25-3p, a 63 bp specific amplicon of miR-16-5p and a 105 bp specific amplicon of RNU48, respectively.

The 7500 Real-Time PCR System (Applied Biosystems, Carlsbad, CA, USA) was used for the qPCR assays of CD138+ miR-25 levels. The 10 μL reactions included Kapa SYBR^®^ Fast Universal 2X qPCR Master Mix (Kapa Biosystems, Inc., Woburn, MA, USA), 200 nM of each PCR primer and 1 ng of cDNA template. The thermal protocol consisted of 95 °C for 3 min for polymerase activation, followed by 40 cycles of denaturation step at 95 °C for 15 s and primer annealing/elongation phase at 60 °C for 1 min. Following amplification, melting curve analysis followed by agarose gel electrophoresis was performed to evaluate the reaction specificity. Duplicated reactions were held for the reproducibility of the results while the 2^−ΔΔCT^ relative quantification (RQ) method was utilized for miR-25 expression level analysis, using RNU48 as endogenous reference control for normalization.

The quantification of plasma miR-25 levels was conducted using the QuantStudio^™^ 5 Real-Time PCR System (Applied Biosystems). The reactions of PCR assays were settled as the above, using 0.5 µL of plasma cDNA template, with a modified thermal protocol. More specifically, the activation of polymerase took place at 95 °C for 3 min, while denaturation, primer annealing and elongation at 95 °C for 3 s and 60 °C for 30 s for 40 cycles. All reactions were performed in duplicates while the expression levels of circulating miR-25 were analyzed with the 2^−ΔΔCT^ RQ method, utilizing miR-16 as reference control for normalization purposes.

### Statistical analysis

Statistical analysis was conducted using IBM SPSS Statistics 20 software (IBM Corp., Armonk, New York, USA). The normal distribution of the data was tested using Sapiro–Wilk and Kolmogorov–Smirnov tests. Due to the non-canonical distribution, the correlation of miRNA levels with categorical clinicopathological data was evaluated with Mann–Whitney* U* test and Kruskal–Wallis test. ROC curve analysis of CD138+ miR-25 expression was performed for the discrimination of MM from sMM patients. *p* value was calculated by Hanley and McNeil method. Kaplan–Meier curves using log-rank test and Cox proportional regression analysis were assessed for survival analysis. X-tile algorithm was applied for the adoption of optimal cut-off values of miR-25 levels. Bootstrap Cox proportional regression analysis (1000 bootstrap samples) was applied for internal validation. Finally, the clinical benefit of miR-25 in patients’ prognosis and treatment outcome was evaluated by decision curve analysis (DCA), according to Vickers et al*.* [32], by STATA 13 software (StataCorp LLC, College Station, TX, USA).

## Results

### miRNA-seq profiling of CD138+ plasma cells

**Fig. 2:**
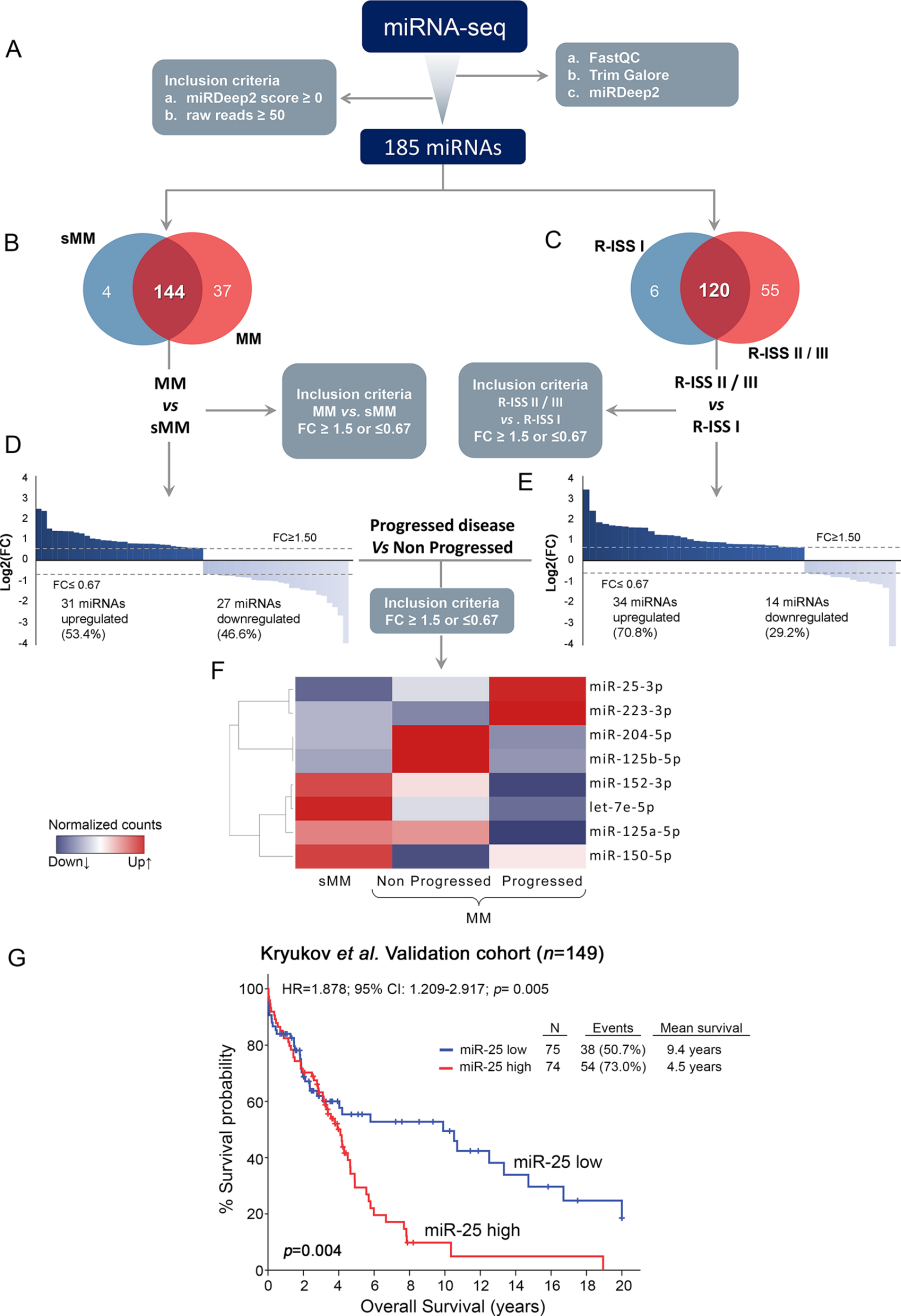
miRNA-seq of CD138+ plasma cells and MM prognosis. **A** Flow diagram of miRNA-seq analysis of CD138+ plasma cells in MM and sMM patients, **B**, **C** Venn diagrams depicting shared miRNAs in **B** MM *vs.* sMM and **C** R-ISS I *vs.* R-ISS II/III patients. **D**, **E** Bar graphs of miRNAs log2FC in **D** MM *vs.* sMM and **E** R-ISS II/III *vs.* R-ISS I stages. **F** Heatmap of the mutually deregulated miRNAs in a. MM *vs.* sMM, b. R-ISS II/III vs. R-ISS I and c. progressed *vs*. non-progressed patients displaying FC ≥ 1.5 or ≤ 0.67. Colorgram illustrates high (red) and low (blue) miRNA expression levels. **G** Kaplan–Meier survival curves of Kryukov et al. MM cohort, according to CD138+ miR-25-3p expression. *p*-value calculated by log-rank test. FC: fold change

To investigate miRNAs differential expression in MM, miRNA˗seq was performed in 24 sMM and MM CD138+ plasma cells. The principal miRNA-seq workflow is schematically presented in Fig. 2. After excluding noise using FastQC, TrimGalore and miRDeep2, 1800 known mature miRNAs were successfully mapped on human genome sequence (hg38). To further filter-out false-positive signals, we excluded miRNAs with negative miRDeep2 score and < 50 raw read counts, narrowing down to 185 mature miRNAs that were selected for downstream analysis (Fig. 2A).

Concerning the differences between MM *vs.* sMM samples, 144 miRNAs were detected in both MM and sMM patients (Fig. 2B). Among them, 58 miRNAs were differentially expressed (31 upregulated and 27 downregulated) between MM and sMM with a fold change (FC) ≥ 1.5 or ≤ 0.67 (Fig. 2D and Additional file 1: Table S1). Focusing on miRNAs alterations between R-ISS stages, 120 miRNAs were found to be concurrently expressed in R-ISS II/III and R-ISS I patients (Fig. 2C), while 34 miRNAs were found to be upregulated and 14 miRNAs downregulated in the advance disease stages, respectively (FC ≥ 1.5 or ≤ 0.67; Fig. 2E and Additional file 2: Table S2). Finally, we examined miRNAs deregulation concerning disease progression (relapse and/or death). Among the miRNAs mutually deregulated in MM *vs.* sMM and R-ISS II/III *vs.* R-ISS I patients, 8 miRNAs (miR-25-3p, miR-223-3p, miR-204-5p, miR-125b-5p, miR-152-3p, let-7e-5p, miR-125a-5p, miR-150-5p) displayed differential expression levels (FC ≥ 1.5 or ≤ 0.67) between progressed and non-progressed patients (Fig. 2F and Additional file 3: Table S3).

These 8 miRNAs were selected for in silico analysis and clinical evaluation in the Kryukov et al. cohort. Τhe survival analysis highlighted miR-25-3p (miR-25) to be the only candidate displaying significant association (unfavorable) with OS [Hazard Ratio (HR) = 1.878; 95% Confidence Intervals (CI): 1.209–2.917; p = 0.005] of MM patients (Fig. 2G and Additional file 4: Figure S1A), while no statistically significant correlation was documented for the other candidates (Additional file 4: Figure S1).

**Fig. 3:**
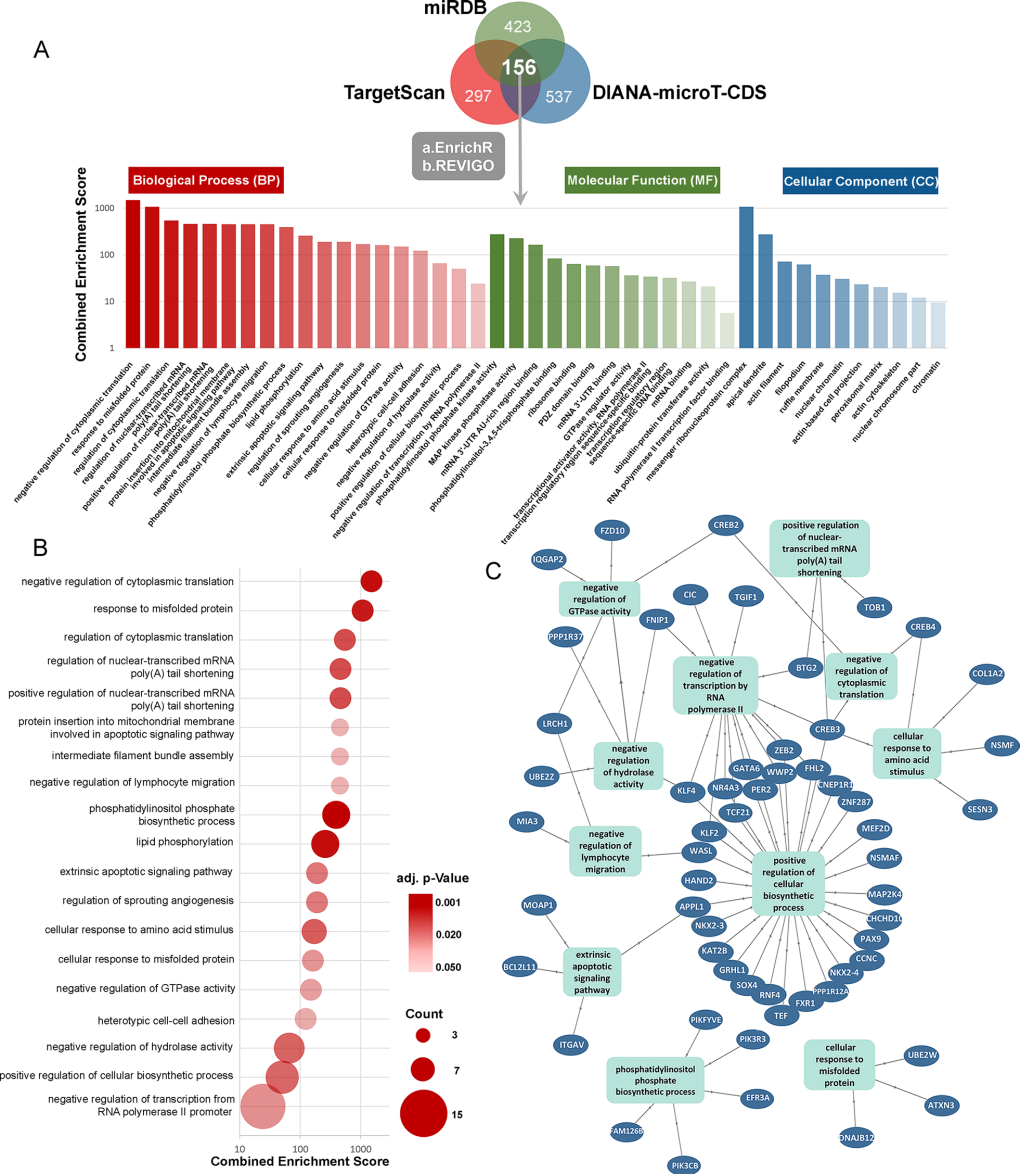
Gene Ontology (GO) enrichment analysis of miR-25. **A** Top significant GO terms associated with miR-25 target genes. The vertical axis represents the combined enrichment score, and the horizontal axis represents the GO category. **B** Dot plot showing enrichment of GO biological processes. The color of the dots represents the *p*-value and the size represents the number of the miR-25 target genes associated with the BP term. **C** Association between miR-25 target genes and statistically significant GO BP terms, through GOnet tool

Furthermore, we assessed target-prediction and GO enrichment analysis to gain insight into the biological and functional role of miR-25. Identification of the potential target genes of miR-25 was carried out through miRDB, TargetScanHuman 8.0 and DIANA TOOLS-microT-CDS target prediction tools. Target prediction analysis revealed 156 overlapping genes as potential direct miR-25 targets, while GO analysis unveiled 19 BPs, 11 CCs and 13 MFs significantly enriched (Fig. 3). Among the top ten BPs enriched, miR-25 was predicted to be involved in cancer associated pathways/processes including, cytoplasmic translation, lymphocyte migration and apoptotic signaling. These findings prompted us to further evaluate miR-25 clinical significance in improving risk-stratification and treatment prognosis of MM patients in our screening cohort (n = 167).

### CD138+ overexpression of miR-25 is associated with inferior survival outcome and short-term progression

miR-25 levels and distribution in our screening cohort are displayed in Figure S2 (Additional file 5: Figure S2). Kaplan–Meier and Cox regression analyses were assessed, utilizing disease progression (relapse and/or death; whichever came first) and patients’ mortality as clinical endpoint events for the PFS and OS, respectively. Using the X-tile algorithm, the 55th percentile of miR-25 levels was adopted as the optimal cut-off value classifying MM patients to “miR-25-high” and “miR-25-low”.

**Fig. 4:**
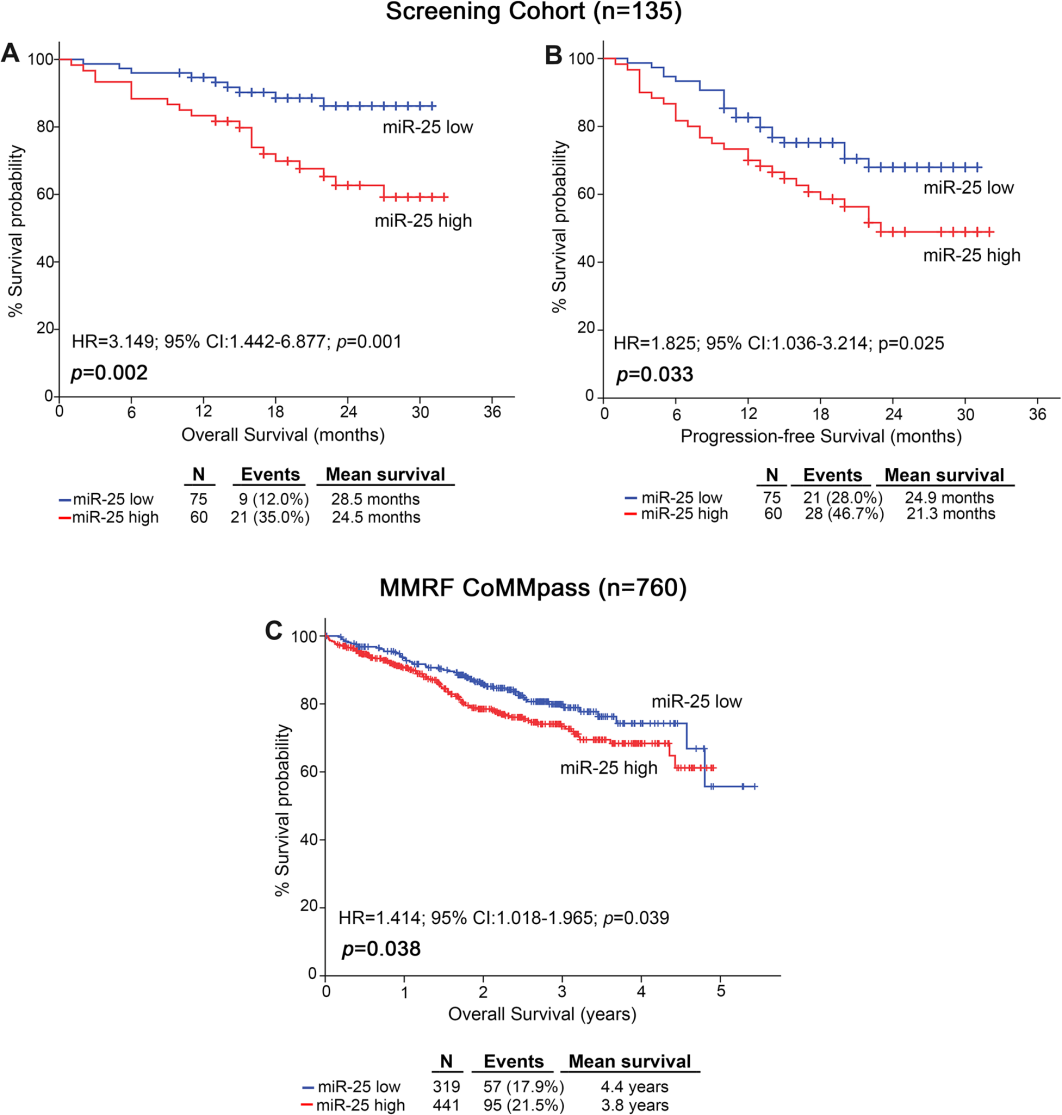
miR-25 overexpression in CD138+ plasma cells is associated with poor overall and progression-free survival. Kaplan–Meier survival curves for **A** overall survival (OS) and **B** progression-free survival (PFS) of the screening cohort, and for **C** OS of the MMRF CoMMpass validation cohort, according to CD138+ miR-25 expression. *p*-values calculated by log-rank test

**Fig. 5:**
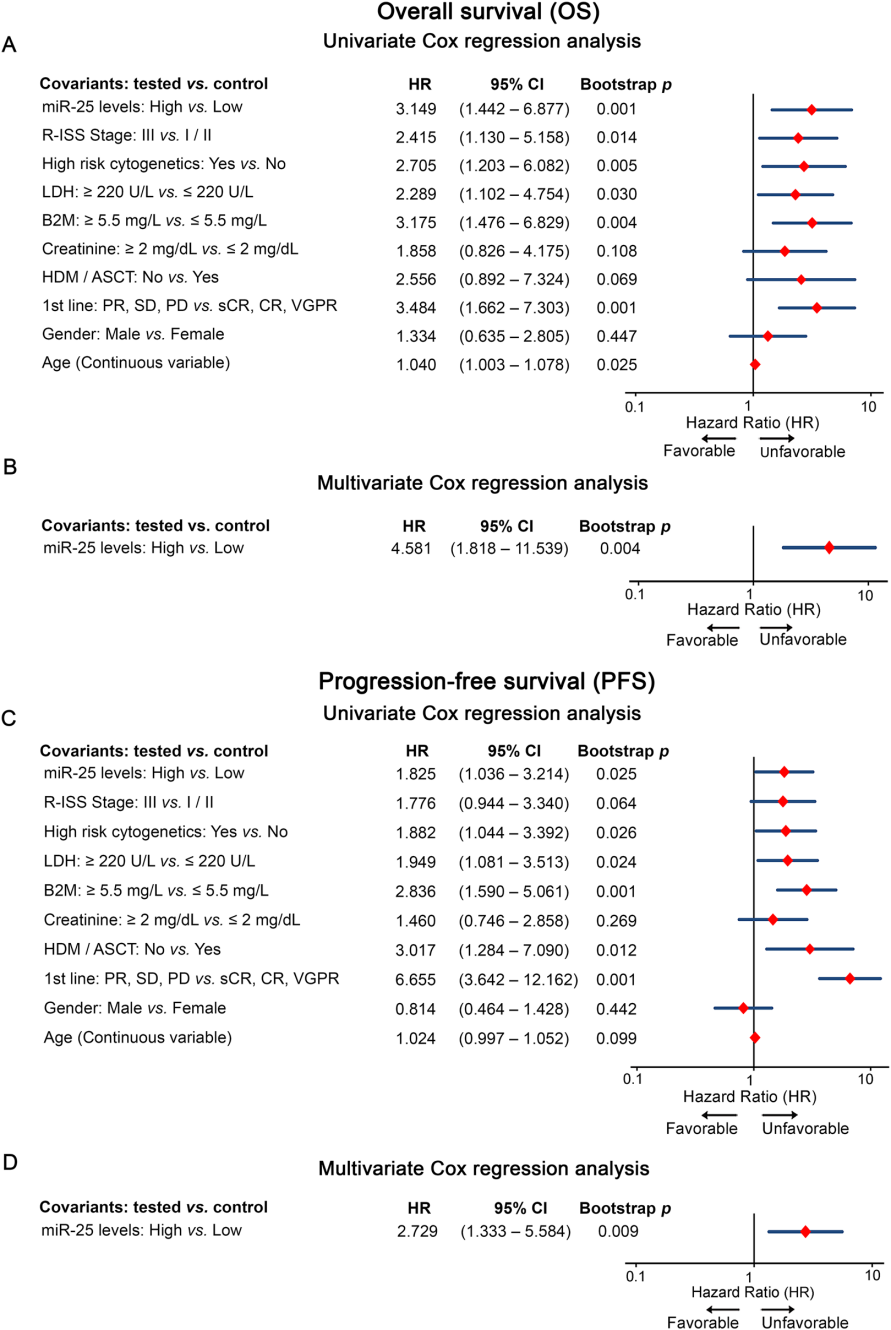
CD138+ overexpression of miR-25 independently predicts MM post-treatment outcome. Forest plots of the univariate **A**, **C** and multivariate **B**, **D** Cox proportional regression analyses. Multivariate analysis adjusted for CD138+ miR-25 levels, R-ISS stage, high-risk cytogenetics [t(4;14) del (17p13), t(14;16), t(11;14), del(13q)], B2M, LDH and creatinine levels, gender, age, HDM/ASCT and response to 1st line therapy. Internal validation was performed by bootstrap analysis based on 1000 bootstrap samples. HR: Hazard Ratio, 95% CI: 95% confidence interval of the estimated HR

The survival analysis of our screening cohort highlighted the unfavorable nature of miR-25 overexpression for MM prognosis, confirming miRNA-seq and Kryukov et al. cohort analysis. More precisely, Kaplan–Meier curves depicted the significantly shorter OS (p = 0.002, Fig. 4A) and PFS (p = 0.033; Fig. 4B) of MM patients with CD138+ overexpression of miR-25 compared to “miR-25-low” group. The adverse prognostic utility of miR-25 overexpression was maintained concerning MM-specific survival (p = 0.001; Additional file 6: Figure S3). Univariate Cox regression analysis confirmed the poor survival (HR = 3.149; 95%CI 1.442–6.877; p = 0.001; Fig. 5A) and the higher risk for post-treatment progression (HR = 1.825; 95%CI 1.036–3.214; p = 0.025; Fig. 5C) of the MM patients with increased miR-25 levels. To further reinforce our findings, the MMRF CoMMpass study was used as a second institutional-independent validation cohort. In line with our findings, Kaplan–Meier curve (p = 0.038; Fig. 4C) and Cox analysis (HR: 1.414; 95% CI 1.018–1.965; p = 0.039) of MMRF CoMMpass study clearly confirmed the powerful prediction value of miR-25 and the significantly worse post-treatment outcome of the “miR-25-high” group. Finally, multivariate Cox regression models adjusted for CD138+ miR-25 levels, R-ISS stage, high risk cytogenetics [t(4;14) del (17p13), t(14;16), t(11;14), del(13q)], LDH, B2M, creatinine, HDM/ASCT, response to 1st line therapy, gender and age (Fig. 5B, D and Additional file 7: Table S4), illustrated the independent prognostic value for poor OS (HR = 4.581; 95%CI 1.818–11.539; p = 0.004; Fig. 5B) and short-term post-treatment progression (HR = 2.729; 95%CI 1.333–5.584; p = 0.009; Fig. 5D) of miR-25 overexpression in CD138+ plasma cells. Focusing on the expression of CD138+ miR-25 in benign disease states, the analysis did not reveal significant differences of miR-25 levels between sMM and MM patients (p = 0.083; Additional file 8: Figure S4).

### Εvaluation of miR-25 improves risk-stratification by the clinically established disease markers

**Fig. 6:**
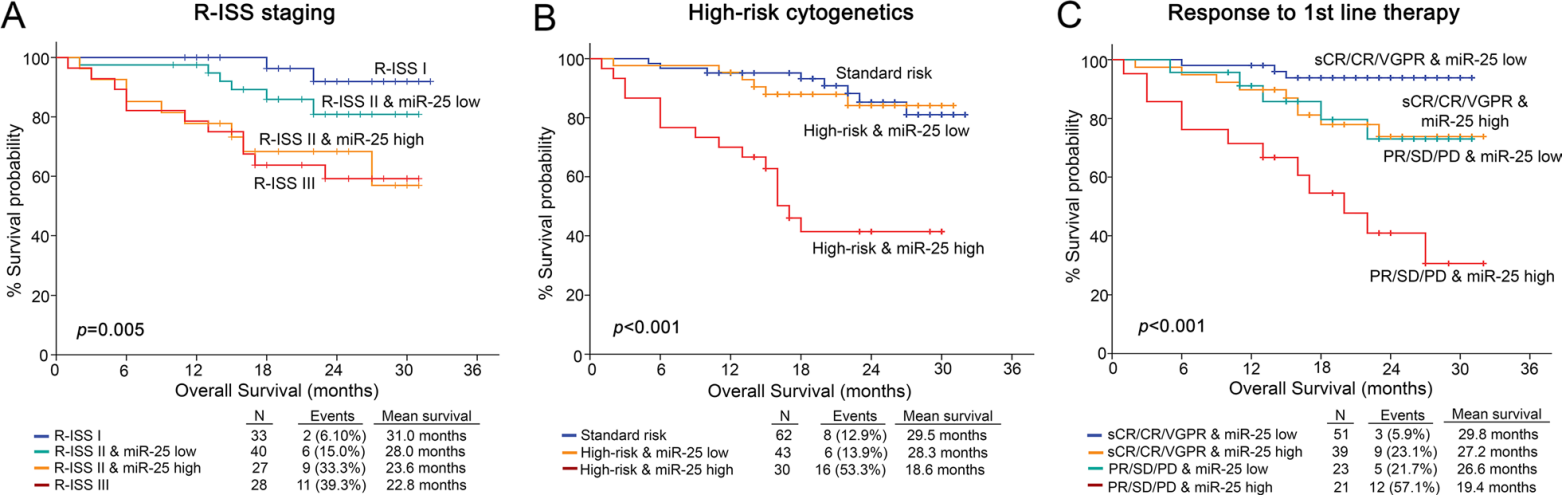
Evaluation of CD138+ miR-25 levels improves risk-stratification and results to superior clinical benefit in MM prognosis. Kaplan–Meier survival curves for the overall survival (OS) of MM patients according to CD138+ expression of miR-25 in combination with R-ISS stage **A**, high-risk cytogenetics **B** and response to 1st line therapy **C**. *p*-values calculated by log-rank test. sCR: stringent complete response, CR: complete response and VGPR: very good partial response, PR: partial response, SD: stable disease, PD: progressive disease

Motivated by the independent prognostic value of miR-25, we investigated its ability to improve disease prognosis by the established disease markers of “R-ISS stage”, “high-risk cytogenetics” and “response to 1st line therapy” (IMWG guidelines). In this regard, the incorporation of miR-25 expression with disease markers unveiled a powerful risk-stratification strategy for MM treatment outcome (Fig. 6 and Additional file 9: Figure S5).

More precisely, integration of miR-25 with R-ISS stage resulted in a superior risk-stratification of R-ISS II patients, with those overexpressing miR-25 to suffer from significantly worse survival, analogous to R-ISS III patients (p = 0.005; Fig. 6A). Similarly, miR-25 expression was able also to stratify high-risk cytogenetics group, as high-risk patients with elevated miR-25 levels reported remarkably poorer OS (p < 0.001; Fig. 6B) and PFS (p < 0.001; Additional file 9: Figure S5B) compared to “miR-25-low” subgroup, that resembled the standard-risk patients. Finally, the response to 1st line therapy represents one of the most efficient and accurate markers for MM risk-stratification and adjustment of treatment decisions, and the concurrent evaluation of CD138+ miR-25 greatly ameliorated its predictive strength. More precisely, patients with optimal responses (sCR, CR, VGPR) and miR-25 overexpression, presented significantly worse OS (p < 0.001; Fig. 6C) and PFS (p < 0.001; Additional file 9: Figure S5C) compared to “miR-25-low” optimal responders. In addition, the elevated miR-25 levels could also efficiently define the patients with unfavorable 1st line response (PR/SD/PD) at higher risk for poor OS and PFS.

**Fig. 7:**
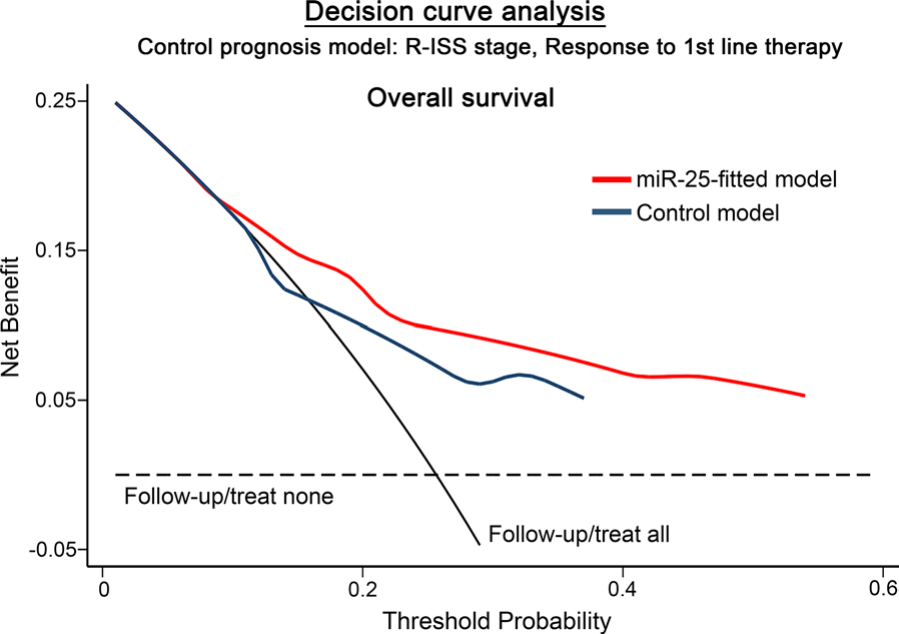
Decision curve analysis (DCA) underlines the superior clinical benefit of miR-25-fitted multivariate prognostic models. DCA curves of “miR-25-fitted” and “control” multivariate prognostic models for patients’ OS. Net benefit is plotted against various ranges of threshold probabilities

Finally, DCA was performed according to Vickers et al. to evaluate the net benefit in MM prognosis of multivariate models incorporating CD138+ miR-25 levels with the clinically used markers of R-ISS and response to 1st line therapy. DCA curve clearly demonstrated the augmented clinical net benefit of the miR-25-fitted models for OS prognosis, compared to the control model of the clinical markers alone, highlighting the superior risk-stratification provided by miR-25 evaluation of CD138+ plasma cells (Fig. 7).

### Circulating miR-25 levels are correlated with poor post-treatment outcome in MM

**Fig. 8:**
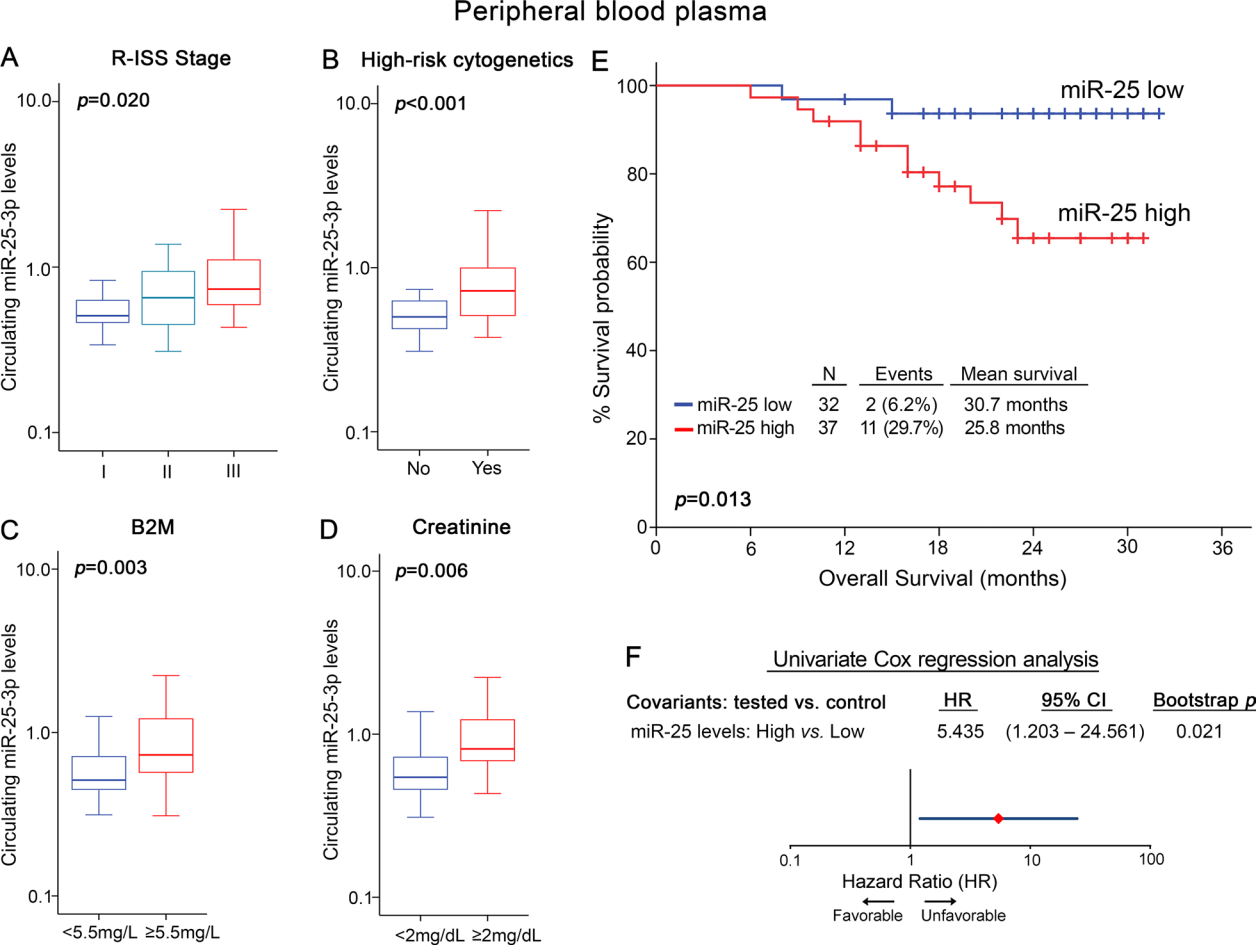
Circulating miR-25 is associated with aggressive disease phenotype and poor survival. **A**–**D** Box plots presenting the correlation of plasma miR-25 levels with R-ISS stage **A**, high risk cytogenetics **B** B2M levels **C** and creatinine levels **D** in the screening MM cohort. p-values calculated by Kruskal–Wallis test **A** and Mann–Whitney *U* test **B**–**D**. **E** Kaplan–Meier survival curves for overall survival (OS) of the MM screening cohort. *p*-value calculated by log-rank test. Forest plot of the univariate **F** Cox proportional regression analysis. Internal validation was performed by bootstrap analysis based on 1000 bootstrap samples. HR: Hazard Ratio, 95% CI: 95% confidence interval of the estimated HR

To investigate miR-25 ability to serve as non-invasive prognostic marker in MM, miR-25 levels were quantified in pre-treatment peripheral plasma samples of our screening cohort (Fig. 8, Additional file 10: Table S5). The analysis highlighted the strong association of circulating miR-25 levels with the aggressive disease phenotype and poor treatment outcome. More precisely, higher plasma miR-25 levels were associated with advance R-ISS stages (p = 0.020; Fig. 8A), high-risk cytogenetics (p < 0.001; Fig. 8B), elevated B2M (p = 0.003; Fig. 8C) and creatinine levels (p = 0.006; Fig. 8D). Moreover, Kaplan–Meier curves (p = 0.013; Fig. 8E) and univariate Cox regression analysis (HR = 5.435; 95%CI 1.203–24.561; p = 0.021; Fig. 8F and Additional file 10: Table S5), highlighted the significantly shorter OS of the “circulating miR-25-high” group compared to MM patients with lower miR-25 plasma levels at disease diagnosis. The survival analysis did not highlight any statistically significant correlation of circulating miR-25 levels with patients’ PFS (Additional file 11: Figure S6).

## Discussion

Although significant advancements have been made in MM treatment and monitoring in recent years, the high relapse and treatment resistance propensity remain the major obstacles for optimal disease management. Hence, the exploitation of new tools to ameliorate patients’ prognosis and prediction of treatment outcome is an urgent demand for translational research. In this context, currently comprehensive studies have focused their attention on shedding light on MM pathogenesis and biomarker establishment.

Minimal residual disease (MRD) is associated with significantly shorter OS expectancy and considered as a potent prognostic factor in MM, improving patients’ risk stratification and clinical management [23, 33]. Moreover, both the elevated serum levels of the angiogenic factors FGF-2 and VEGF, and the chemokine CCL18 are correlated with worse patients’ outcome [34, 35]. Likewise, ITG6 downregulation was characterized as an unfavorable prognostic indicator, displaying strong correlation with shorter survival outcome, and with plasma cell leukemia (PCL) phenotype [36]. Furthermore, mounting evidence supports that miRNAs dysregulation, have been strongly correlated with early disease progression, poor prognosis and treatment resistance [37]. More specifically, overexpression of miR-181a, miR-410 and miR-105 in CD138+ plasma cells have been associated with poor patients’ outcome, while downregulation of miR-137 was highlighted as unfavorable prognostic marker [18, 38–40]. Concerning treatment response, upregulation of miR-221/222 was associated with dexamethasone resistance [41].

In the present study, miRNA-seq of CD138+ plasma cells unveiled 8 miRNAs (miR-25-3p, miR-223-3p, miR-204-5p, miR-125b-5p, miR-152-3p, let-7e-5p, miR-125a-5p, and miR-150-5p) to be altered between MM *vs* sMM, R-ISS II/III *vs* R-ISS I and progressed *vs* non-progressed MM patients. Kryukov et al*.* validation cohort was used to analyze the clinical significance of these miRNAs, highlighting miR-25 for further in-depth clinical evaluation in our screening and MMRF CoMMpass validation cohort.

The analysis of our screening cohort documented that CD138+ miR-25 upregulation is significantly associated with higher risk for inferior survival and short-term disease progression of MM patients, independently of the established MM markers and patients’ clinicopathological data. Notably, the analysis of MMRF CoMMpass study clearly confirmed the worse outcome of the patients overexpressing miR-25. Moreover, multivariate prognostic models demonstrated the ability of miR-25 to ameliorate the clinical routine of the established MM prognostic indicators, including R-ISS stage, high-risk cytogenetics, and response to 1st line therapy. In this context, the evaluation of CD138+ miR-25 levels significantly improved the risk-stratification of MM patients, resulting in the advanced positive prediction of patients’ poor treatment outcome within R-ISS II, high-risk cytogenetics and optimal treatment responders (sCR, CR, VGPR) subgroups. Consequently, DCA demonstrated that miR-25-fitted multivariate models led to a superior MM prognostication.

miR-25 is encoded by *MIR25* gene (7q22.1) and belongs to the highly conserved miR-106b-25 cluster (miR-106b, miR-93 and miR-25), a widely known oncogenic cluster [42, 43]. Our findings are in line with previous studies, supporting miR-25 oncogenic nature in MM. Pichiorri et al. were the first to report a significant upregulation of miR-25 levels in BM plasma cells of MM patients compared to healthy individuals, while Zhou et al*.* reported its elevation in high-risk myeloma stages [44, 45]. Mechanistically, miR-25 suppresses p53 signaling, and thus inhibits apoptosis and cell senescence, by targeting directly *TP53* expression and indirectly by silencing histone acetyltransferase PCAF, a potent coactivator of p53 [44, 46, 47]. In addition, miR-25-targeting of PTEN has been demonstrated in vitro to activate PI3K/AKT pathway, resulting in MM proliferation and apoptosis attenuation, while MYC-induced overexpression of miR-25 was documented to mediate dexamethasone resistance in MM via targeting ULK1 and p27 [48, 49].

Besides MM, miR-25 has been documented to promote triple-negative breast cancer and glioma progression through the activation of AKT/MAPK-ERK pathway and the suppression of PTEN, respectively [50, 51] while in lung cancer, miR-25 facilitates lymph node metastasis by targeting CDH1, and correlates with significantly worse patients’ outcome [52]. Moreover, miR-25 overexpression has been associated with tumor progression and poor prognosis in colorectal cancer (CRC) [53], while exosomal miR-25 has been documented to induce macrophage M2 polarization, via PTEN targeting and PI3K/ACT activation, promoting EMT, angiogenesis and tumor metastasis in CRC [54, 55]. Finally, miR-25 has been reported to stimulate growth and invasion of ovarian cancer cells by targeting key tumor-suppressors, including LATS2 and BIM [56, 57], while miR-25 overexpression in ovarian cancer patients has been associated with adverse disease outcome [58]. Finally, high levels of miR-25 have been significantly correlated with poor outcome of pancreatic and hepatocellular cancer patients, highlighting its potent clinical prognostic impact [59, 60].

BM biopsies remain the gold standard clinical tool for MM prognostication. However, due to the clonal patchy distribution of plasma cells, BM biopsies may not fully reflect disease heterogeneity, while submitting patients to invasive procedures. In this regard, liquid biopsies hold promise as new minimally invasive tools, and due to their small size and high stability, circulating miRNAs have emerged as modern non-invasive molecular markers [61, 62]. Notably, recent studies have highlighted the prognostic value of circulating miR-25 in the highly prevalent colorectal and lung carcinomas [63, 64]. Given the potent clinical utility of CD138+ miR-25 levels, we further evaluated its significance in patients’ pre-treatment peripheral plasma. In this regard, higher miR-25 plasma levels were associated with advance R-ISS stages, high-risk cytogenetics, and increased B2M and creatinine levels, as well as with worse post-treatment survival, endorsing its promise for modern non-invasive molecular diagnostics.

The moderately short median follow-up time (24 months) of our screening cohort constitutes the major limitation of our study, while undoubtedly, longer follow-up could give a more insightful association between miR-25 levels and MM patients’ survival. Moreover, the available blood samples to clinically evaluate circulating miR-25 were limited. However, our results clearly highlighted the strong correlation of circulating miR-25 with poor post-treatment outcome in MM, and future large-scale studies could intensify our findings, supporting circulating miR-25 as a novel non-invasive predictor in MM. Finally, although the validation of the prognostic potential of CD138+ miR-25 levels by three independent patient cohorts, strongly supports its impact on MM prognostication, further multi-institutional validation will define the optimal cut-off values and procedures to be utilized in clinical setting.

## Conclusions

In conclusion, CD138+ miR-25 overexpression was detected by miRNA-seq in MM compared to sMM, in R-ISS II/III, and in post-treatment progressed patients. The analysis of three independent MM cohorts highlighted that miR-25 overexpression can serve as an independent molecular predictor of worse disease survival and short-term progression of the patients following treatment. Moreover, miR-25-fitted multivariate models offered superior risk-stratification of the patients and improved prediction of post-treatment outcome compared to the clinically used disease markers. Finally, we have demonstrated that elevated pre-treatment plasma miR-25 is associated with advance disease stages and poor survival of MM patients following treatment, serving as potent non-invasive molecular tool in ameliorating MM patients’ risk-stratification and prognosis.

## Supplementary Information

**Additional file 1: Table S1.** Deregulated miRNAs in MM *vs *sMM by miRNA-seq.

**Additional file 2: Table S2.** Deregulated miRNAs in R-ISS II / III *vs* R-ISS I by miRNA-seq.

**Additional file 3: Table S3.** Mutually deregulated miRNAs in a. MM *vs*. sMM, b. R-ISS II / III *vs*. R-ISS I and c. progressed *vs.* non-progressed patients by miRNA-seq.

**Additional file 4: Figure S1.** Kaplan-Meier survival curves for the overall survival (OS) of the Kryukov *et al.* MM validation cohort according to miRNAs of interest. (A) miR-25, (B) let-7e, (C) miR-152, (D) miR-204, (E) miR-125a, (F) miR-125b, (G) miR-223, (H) miR-150. *p* values calculated by log-rank test.

**Additional file 5: Figure S2.** Descriptive statistics of miR-25 in the MM screening cohort. (A) miR-25 distribution in CD138+ plasma cells, (B) miR-25 distribution in plasma samples.

**Additional file 6: Figure S3.** Kaplan-Meier survival curves for the cancer-specific survival (CSS) of the MM patients according to CD138+ miR-25 levels. *p* values calculated by log-rank test.

**Additional file 7: Table S4.** Cox regression analysis for the prediction of MM patients’ risk for death (OS) and progression (PFS) based on CD138+ miR-25 levels.

**Additional file 8: Figure S4.** Analysis of miR-25 between sMM and MM patients. (A) Box plot presenting miR-25 levels in MM compared to sMM patients. *p *value calculated by Mann-Whitney U test, (B) ROC curve analysis of miR-25 levels for the discrimination of MM patients from sMM. AUC: area under the curve, 95% CI: 95% confidence intervals. *p* value calculated by the Hanley and McNeil method.

**Additional file 9: Figure S5.** Kaplan-Meier survival curves for the progression-free survival (PFS) of MM patients according to CD138+ miR-25 levels combined with (A) R-ISS stage, (B) high-risk cytogenetics and (C) response to 1st line therapy. *p* values calculated by log-rank test. sCR: stringent complete response, CR: complete response, VGPR: very good partial response, PR: partial response, SD: stable disease, and PD: progressive disease.

**Additional file 10: Table S5.** Cox regression analysis for the prediction of MM patients’ risk for death (OS) based on circulating miR-25 levels.

**Additional file 11: Figure S6.** Kaplan-Meier survival curves for progression-free survival (PFS) of the MM screening cohort according to circulating miR-25 levels. *p* values calculated by log-rank test.

## Data Availability

All the data are available from the corresponding authors on reasonable request.

## Abbreviations

3’UTR: 3′-Untranslated region
B2M: Beta-2 microglobulin
BM: Bone marrow
BP: Biological process
CC: Cellular component
CR: Complete response
DCA: Decision curve analysis
EDTA: Ethylenediaminetetraacetic acid
FISH: Fluorescence in situ hybridization
FC: Fold change
GDC Data Portal: Genomic data commons data portal
GO: Gene ontology
HDM/ASCT: High dose melphalan therapy with autologous stem cell transplantation
HR: Hazard ratio
IMWG: International myeloma working group
ISS: International staging system
LDH: Lactate dehydrogenase
MF: Molecular function
MGUS: Monoclonal gammopathy of undetermined significance
MM: Multiple myeloma
MMRF: Multiple myeloma research foundation
MRD: Minimal residual disease
NGS: Next generation sequencing
OS: Overall survival
PCL: Plasma cell leukemia
PD: Progressed disease
PFS: Progression free survival
PR: Partial response
REMARK: Reporting recommendations for tumor marker prognostic studies
RISC: RNA-induced silencing complex
R-ISS: Revised international staging system
RQ: Relative quantification
RT-qPCR: Reverse transcription quantitative polymerase chain reaction
sCR: Stringent complete response
SD: Stable disease
sMM: Smoldering multiple myeloma
SNORD48 – RNU48: Small nucleolar RNA C/D box 48
UMI: Unique molecular identifier
VGPR: Very good partial response
