# Supplementary material for: miRNA-seq identification and clinical validation of CD138+ and circulating miR-25 in treatment response of multiple myeloma
Source: J Transl Med. 2023 Apr 6;21:245. doi: 10.1186/s12967-023-04034-5 (PMC10080848; doi:10.1186/s12967-023-04034-5)
Supplement: Supplementary file 1 — Additional file 1: Table S1. Deregulated miRNAs in MM vs sMM by miRNA-seq. [file 12967_2023_4034_MOESM1_ESM.docx]

**Table S1.** Deregulated miRNAs in MM *vs* sMM by miRNA-seq

| **MM vs sMM** | **miRNA** | **miRBase accession** | **Location** | **FC** | **Log2FC** |
| --- | --- | --- | --- | --- | --- |
| **Up** | hsa-miR-424-5p | MIMAT0001341 | Xq26.3 | 5.4315 | 2.4414 |
|  | hsa-miR-204-5p | MIMAT0000265 | 9q21.12 | 5.0576 | 2.3385 |
|  | hsa-miR-486-5p | MIMAT0002177 | 8p11.21 | 2.8325 | 1.5021 |
|  | hsa-miR-194-5p | MIMAT0000460 | 1q41/11q13.1 | 2.6342 | 1.3974 |
|  | hsa-miR-3615 | MIMAT0017994 | 17q25.1 | 2.6258 | 1.3928 |
|  | hsa-miR-342-3p | MIMAT0000753 | 14q32.2 | 2.6061 | 1.3819 |
|  | hsa-miR-34a-5p | MIMAT0000255 | 1p36.22 | 2.5986 | 1.3778 |
|  | hsa-miR-92a-3p | MIMAT0000092 | 13q31.3/Xq26.2 | 2.4939 | 1.3184 |
|  | hsa-miR-141-3p | MIMAT0000432 | 12p13.31 | 2.4193 | 1.2746 |
|  | hsa-miR-138-5p | MIMAT0000430 | 3p21.32/16q13 | 2.2378 | 1.1621 |
|  | hsa-miR-320a-3p | MIMAT0000510 | 8p21.3 | 2.0453 | 1.0323 |
|  | hsa-miR-192-5p | MIMAT0000222 | 11q13.1 | 2.0087 | 1.0063 |
|  | hsa-miR-320c | MIMAT0005793 | 18q11.2 | 1.9322 | 0.9502 |
|  | hsa-miR-140-3p | MIMAT0004597 | 16q22.1 | 1.9192 | 0.9405 |
|  | hsa-miR-320b | MIMAT0005792 | 1p13.1/1q42.11 | 1.8821 | 0.9123 |
|  | hsa-miR-708-5p | MIMAT0004926 | 11q14.1 | 1.8422 | 0.8814 |
|  | hsa-miR-15b-5p | MIMAT0000417 | 3q25.33 | 1.8386 | 0.8786 |
|  | hsa-miR-128-3p | MIMAT0000424 | 2q21.3/ 3p22.3 | 1.7476 | 0.8053 |
|  | hsa-miR-107 | MIMAT0000104 | 10q23.31 | 1.7378 | 0.7973 |
|  | hsa-miR-423-5p | MIMAT0004748 | 17q11.2 | 1.7347 | 0.7947 |
|  | hsa-miR-484 | MIMAT0002174 | 16p13.11 | 1.7279 | 0.7890 |
|  | hsa-miR-339-5p | MIMAT0000764 | 7p22.3 | 1.7244 | 0.7861 |
|  | hsa-miR-223-3p | MIMAT0000280 | Xq12 | 1.7067 | 0.7712 |
|  | hsa-miR-144-3p | MIMAT0000436 | 17q11.2 | 1.6798 | 0.7483 |
|  | hsa-miR-25-3p | MIMAT0000081 | 7q22.1 | 1.6621 | 0.7330 |
|  | hsa-miR-190b-5p | MIMAT0004929 | 1q21.3 | 1.5971 | 0.6754 |
|  | hsa-miR-130b-3p | MIMAT0000691 | 22q11.21 | 1.5655 | 0.6466 |
|  | hsa-miR-221-3p | MIMAT0000278 | Xp11.3 | 1.5420 | 0.6248 |
|  | hsa-miR-23a-3p | MIMAT0000078 | 19p13.12 | 1.5179 | 0.6021 |
|  | hsa-miR-125b-5p | MIMAT0000423 | 11q24.1/ 21q21.1 | 1.5176 | 0.6018 |
|  | hsa-miR-23b-3p | MIMAT0000418 | 9q22.32 | 1.5014 | 0.5863 |
|  | hsa-miR-100-5p | MIMAT0000098 | 11q24.1 | 0.6445 | -0.6338 |
| **Down** | hsa-miR-3613-5p | MIMAT0017990 | 13q14.2 | 0.6443 | -0.6341 |
|  | hsa-miR-125a-5p | MIMAT0000443 | 19q13.41 | 0.6355 | -0.6541 |
|  | hsa-miR-145-5p | MIMAT0000437 | 5q32 | 0.6114 | -0.7099 |
|  | hsa-miR-499a-5p | MIMAT0002870 | 20q11.22 | 0.6065 | -0.7215 |
|  | hsa-miR-30b-5p | MIMAT0000420 | 8q24.22 | 0.6047 | -0.7258 |
|  | hsa-miR-20b-5p | MIMAT0001413 | Xq26.2 | 0.5863 | -0.7703 |
|  | hsa-miR-96-5p | MIMAT0000095 | 7q32.2 | 0.5798 | -0.7864 |
|  | hsa-miR-1277-5p | MIMAT0022724 | Xq24 | 0.5727 | -0.8041 |
|  | hsa-miR-152-3p | MIMAT0000438 | 17q21.32 | 0.5285 | -0.9200 |
|  | hsa-miR-151b | MIMAT0010214 | 14q32.2 | 0.5210 | -0.9407 |
|  | hsa-miR-135a-5p | MIMAT0000428 | 3p21.2/12q23.1 | 0.5189 | -0.9463 |
|  | hsa-miR-183-5p | MIMAT0000261 | 7q32.2 | 0.5169 | -0.9520 |
|  | hsa-miR-627-5p | MIMAT0003296 | 15q15.1 | 0.5086 | -0.9755 |
|  | hsa-let-7e-5p | MIMAT0000066 | 19q13.41 | 0.4864 | -1.0399 |
|  | hsa-miR-182-5p | MIMAT0000259 | 7q32.2 | 0.4709 | -1.0864 |
|  | hsa-miR-330-5p | MIMAT0004693 | 19q13.32 | 0.4064 | -1.2990 |
|  | hsa-miR-10b-5p | MIMAT0000254 | 2q31.1 | 0.4060 | -1.3005 |
|  | hsa-miR-190a-5p | MIMAT0000458 | 15q22.2 | 0.3829 | -1.3848 |
|  | hsa-miR-150-5p | MIMAT0000451 | 19q13.33 | 0.3766 | -1.4091 |
|  | hsa-miR-491-5p | MIMAT0002807 | 9p21.3 | 0.3746 | -1.4165 |
|  | hsa-miR-1307-5p | MIMAT0022727 | 10q24.33 | 0.3255 | -1.6194 |
|  | hsa-miR-548z | MIMAT0018446 | 12q14.2 | 0.3144 | -1.6692 |
|  | hsa-miR-99b-5p | MIMAT0000689 | 19q13.41 | 0.2491 | -2.0052 |
|  | hsa-miR-95-3p | MIMAT0000094 | 4p16.1 | 0.2174 | -2.2013 |
|  | hsa-miR-132-3p | MIMAT0000426 | 17p13.3 | 0.1660 | -2.5906 |
|  | hsa-miR-375-3p | MIMAT0000728 | 2q35 | 0.0667 | -3.9072 |
