# Supplementary material for: miRNA-seq identification and clinical validation of CD138+ and circulating miR-25 in treatment response of multiple myeloma
Source: J Transl Med. 2023 Apr 6;21:245. doi: 10.1186/s12967-023-04034-5 (PMC10080848; doi:10.1186/s12967-023-04034-5)
Supplement: Supplementary file 2 — Additional file 2: Table S2. Deregulated miRNAs in R-ISS II / III vs R-ISS I by miRNA-seq. [file 12967_2023_4034_MOESM2_ESM.docx]

**Table S2.** Deregulated miRNAs in R-ISS II / III *vs* R-ISS I by miRNA-seq

| **R-ISS II / III  vs R-ISS I** | **miRNA** | **miRBase accession** | **Location** | **FC** | **Log2FC** |
| --- | --- | --- | --- | --- | --- |
| **Up** | hsa-miR-125a-5p | MIMAT0000443 | 19q13.41 | 10.2546 | 3.3582 |
|  | hsa-miR-486-5p | MIMAT0002177 | 8p11.21 | 5.1183 | 2.3557 |
|  | hsa-miR-340-5p | MIMAT0004692 | 5q35.3 | 3.5139 | 1.8131 |
|  | hsa-miR-125b-5p | MIMAT0000423 | 11q24.1/ 21q21.1 | 3.3235 | 1.7327 |
|  | hsa-miR-99a-5p | MIMAT0000097 | 21q21.1 | 3.1740 | 1.6663 |
|  | hsa-miR-223-3p | MIMAT0000280 | Xq12 | 3.1080 | 1.6360 |
|  | hsa-miR-152-3p | MIMAT0000438 | 17q21.32 | 3.0534 | 1.6104 |
|  | hsa-miR-150-5p | MIMAT0000451 | 19q13.33 | 2.9659 | 1.5685 |
|  | hsa-let-7c-5p | MIMAT0000064 | 21q21.1 | 2.9566 | 1.5639 |
|  | hsa-miR-143-3p | MIMAT0000435 | 5q32 | 2.9482 | 1.5598 |
|  | hsa-miR-21-5p | MIMAT0000076 | 17q23.1 | 2.6191 | 1.3890 |
|  | hsa-miR-3613-5p | MIMAT0017990 | 13q14.2 | 2.3322 | 1.2217 |
|  | hsa-miR-335-5p | MIMAT0000765 | 7q32.2 | 2.3108 | 1.2084 |
|  | hsa-miR-181b-5p | MIMAT0000257 | 1q32.1/ 9q33.3 | 2.2944 | 1.1981 |
|  | hsa-miR-532-5p | MIMAT0002888 | Xp11.23 | 2.2379 | 1.1622 |
|  | hsa-miR-660-5p | MIMAT0003338 | Xp11.23 | 2.1412 | 1.0984 |
|  | hsa-miR-93-5p | MIMAT0000093 | 7q22.1 | 2.0134 | 1.0096 |
|  | hsa-let-7e-5p | MIMAT0000066 | 19q13.41 | 1.9073 | 0.9316 |
|  | hsa-miR-195-5p | MIMAT0000461 | 17p13.1 | 1.8824 | 0.9125 |
|  | hsa-miR-218-5p | MIMAT0000275 | 4p15.31/ 5q34 | 1.8389 | 0.8788 |
|  | hsa-miR-183-5p | MIMAT0000261 | 7q32.2 | 1.8338 | 0.8749 |
|  | hsa-miR-194-5p | MIMAT0000460 | 1q41/ 11q13.1 | 1.8222 | 0.8657 |
|  | hsa-miR-192-5p | MIMAT0000222 | 11q13.1 | 1.7315 | 0.7920 |
|  | hsa-miR-185-5p | MIMAT0000455 | 22q11.21 | 1.7166 | 0.7795 |
|  | hsa-miR-421 | MIMAT0003339 | Xq13.2 | 1.7130 | 0.7765 |
|  | hsa-miR-18a-5p | MIMAT0000072 | 13q31.3 | 1.7127 | 0.7762 |
|  | hsa-miR-19a-3p | MIMAT0000073 | 13q31.3 | 1.6978 | 0.7637 |
|  | hsa-miR-98-5p | MIMAT0000096 | Xp11.22 | 1.6898 | 0.7569 |
|  | hsa-miR-30a-5p | MIMAT0000087 | 6q13 | 1.6508 | 0.7231 |
|  | hsa-miR-651-5p | MIMAT0003321 | Xp22.31 | 1.6479 | 0.7206 |
|  | hsa-miR-25-3p | MIMAT0000081 | 7q22.1 | 1.5758 | 0.6561 |
|  | hsa-miR-365a-3p | MIMAT0000710 | 16p13.12 | 1.5597 | 0.6412 |
|  | hsa-miR-365b-3p | MIMAT0022834 | 17q11.2 | 1.5597 | 0.6412 |
|  | hsa-miR-96-5p | MIMAT0000095 | 7q32.2 | 1.5250 | 0.6089 |
| **Down** | hsa-miR-628-5p | MIMAT0004809 | 15q21.3 | 0.6677 | -0.5827 |
|  | hsa-miR-320a-3p | MIMAT0000510 | 8p21.3 | 0.6430 | -0.6370 |
|  | hsa-miR-140-3p | MIMAT0004597 | 16q22.1 | 0.6400 | -0.6438 |
|  | hsa-miR-625-3p | MIMAT0004808 | 14q23.3 | 0.6239 | -0.6807 |
|  | hsa-miR-9-5p | MIMAT0000441 | 1q22/ 5q14.3/ 15q26.1 | 0.5887 | -0.7644 |
|  | hsa-miR-141-3p | MIMAT0000432 | 12p13.31 | 0.5859 | -0.7713 |
|  | hsa-miR-551b-3p | MIMAT0003233 | 3q26.2 | 0.5779 | -0.7911 |
|  | hsa-miR-484 | MIMAT0002174 | 16p13.11 | 0.5364 | -0.8987 |
|  | hsa-miR-222-3p | MIMAT0000279 | Xp11.3 | 0.5052 | -0.9852 |
|  | hsa-miR-221-3p | MIMAT0000278 | Xp11.3 | 0.5044 | -0.9874 |
|  | hsa-miR-200c-3p | MIMAT0000617 | 12p13.31 | 0.4754 | -1.0729 |
|  | hsa-miR-10a-5p | MIMAT0000253 | 17q21.32 | 0.4625 | -1.1124 |
|  | hsa-miR-331-3p | MIMAT0000760 | 12q22 | 0.4138 | -1.2730 |
|  | hsa-miR-204-5p | MIMAT0000265 | 9q21.12 | 0.0253 | -5.3060 |
