# Supplementary material for: miRNA-seq identification and clinical validation of CD138+ and circulating miR-25 in treatment response of multiple myeloma
Source: J Transl Med. 2023 Apr 6;21:245. doi: 10.1186/s12967-023-04034-5 (PMC10080848; doi:10.1186/s12967-023-04034-5)
Supplement: Supplementary file 3 — Additional file 3: Table S3. Mutually deregulated miRNAs in a. MM vs. sMM, b. R-ISS II / III vs. R-ISS I and c. progressed vs. non-progressed patients by miRNA-seq. [file 12967_2023_4034_MOESM3_ESM.docx]

**Table S3.** Mutually deregulated miRNAs in a. MM *vs*. sMM, b. R-ISS II / III *vs*. R-ISS I and c. progressed *vs.* non-progressed patients by miRNA-seq

| **Progressed vs non-progressed** | **miRNA** | **miRBase accession** | **Location** | **FC** | **Log2FC** |
| --- | --- | --- | --- | --- | --- |
| **Up** | hsa-miR-223-3p | MIMAT0000280 | Xq12 | 7.9435 | 2.9898 |
|  | hsa-miR-150-5p | MIMAT0000451 | 19q13.33 | 2.8695 | 1.5208 |
|  | hsa-miR-25-3p | MIMAT0000081 | 7q22.1 | 1.5619 | 0.6433 |
| **Down** | hsa-let-7e-5p | MIMAT0000066 | 19q13.41 | 0.6746 | -0.5678 |
|  | hsa-miR-152-3p | MIMAT0000438 | 17q21.32 | 0.4933 | -1.0194 |
|  | hsa-miR-125b-5p | MIMAT0000423 | 11q24.1/ 21q21.1 | 0.4218 | -1.2454 |
|  | hsa-miR-125a-5p | MIMAT0000443 | 19q13.41 | 0.4083 | -1.2922 |
|  | hsa-miR-204-5p | MIMAT0000265 | 9q21.12 | 0.0849 | -3.5568 |
