# Supplementary figures and images for: miRNA-seq identification and clinical validation of CD138+ and circulating miR-25 in treatment response of multiple myeloma
Source: J Transl Med. 2023 Apr 6;21:245. doi: 10.1186/s12967-023-04034-5 (PMC10080848; doi:10.1186/s12967-023-04034-5)

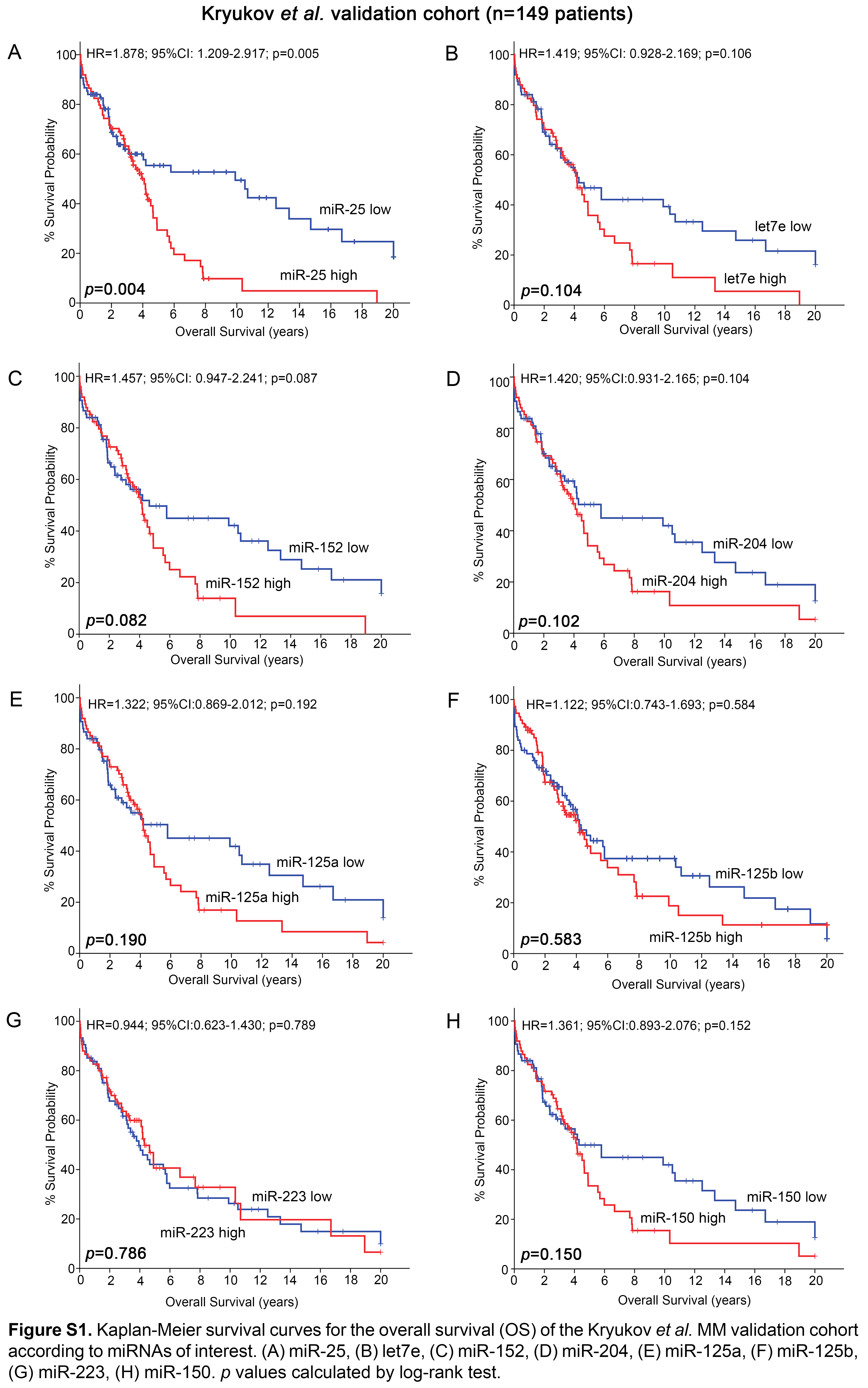

Supplement: Supplementary file 4 — Additional file 4: Figure S1. Kaplan-Meier survival curves for the overall survival (OS) of the Kryukov et al. MM validation cohort according to miRNAs of interest. (A) miR-25, (B) let-7e, (C) miR-152, (D) miR-204, (E) miR-125a, (F) miR-125b, (G) miR-223, (H) miR-150. p values calculated by log-rank test. [file 12967_2023_4034_MOESM4_ESM.tif]

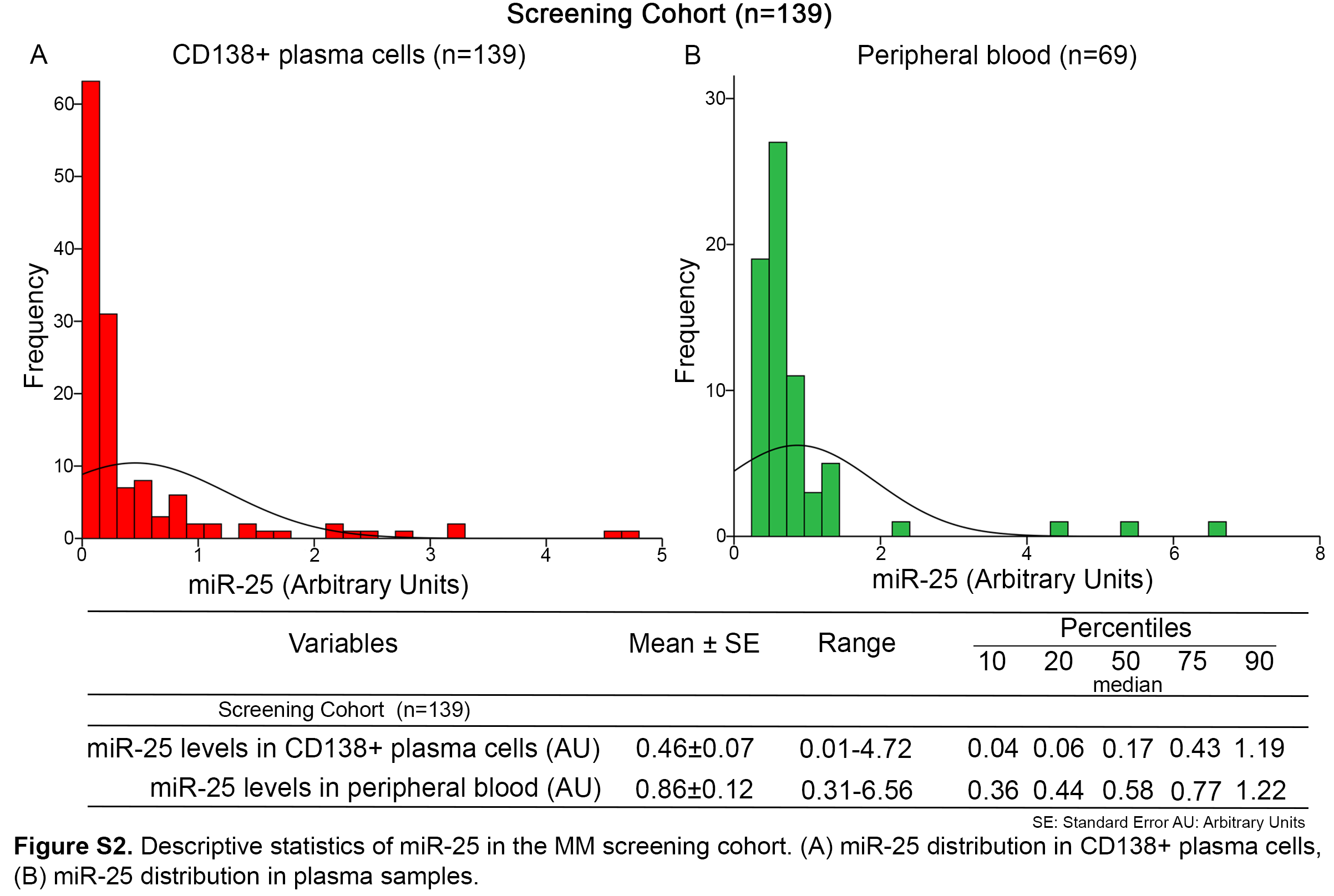

Supplement: Supplementary file 5 — Additional file 5: Figure S2. Descriptive statistics of miR-25 in the MM screening cohort. (A) miR-25 distribution in CD138+ plasma cells, (B) miR-25 distribution in plasma samples. [file 12967_2023_4034_MOESM5_ESM.tif]

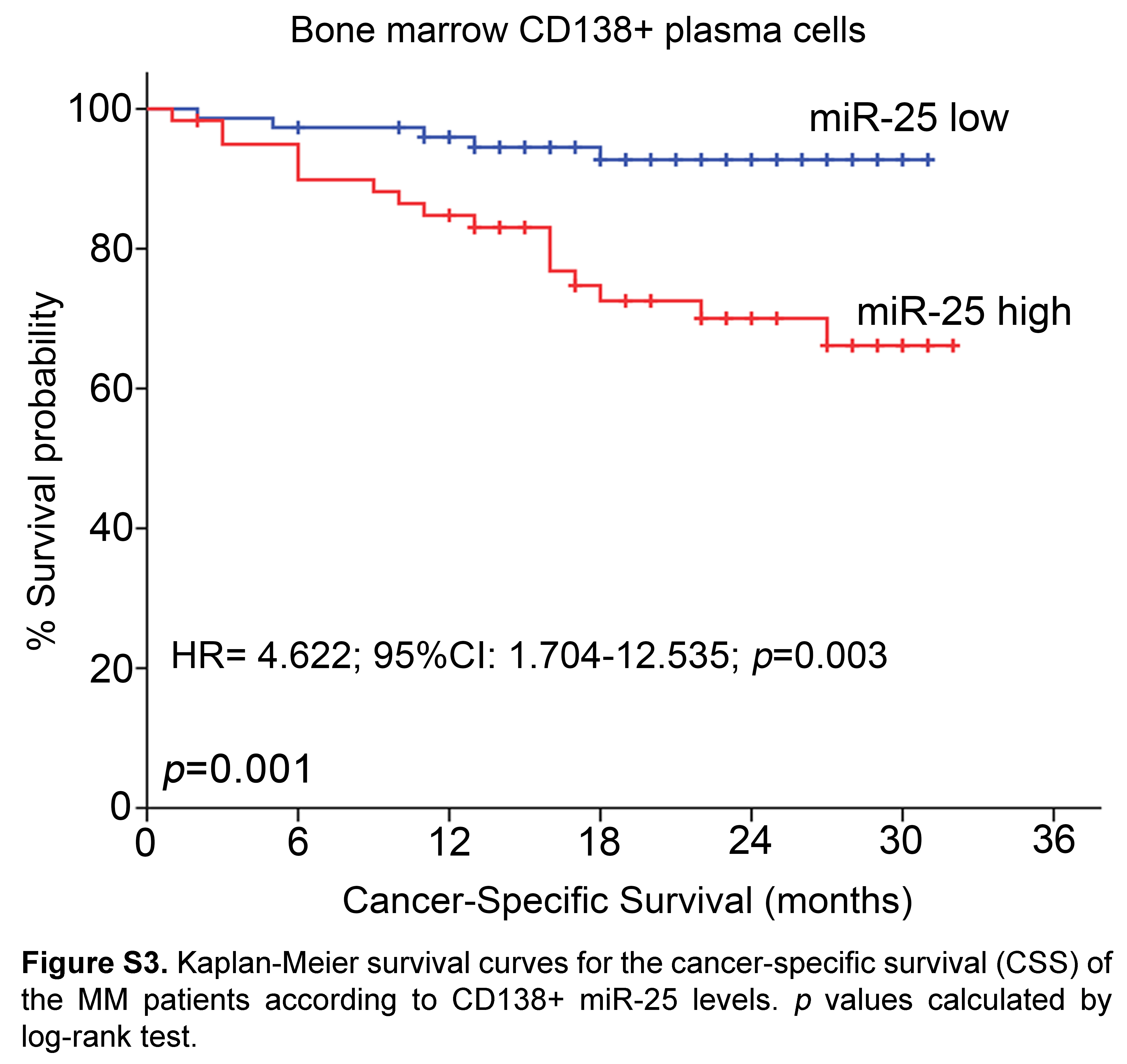

Supplement: Supplementary file 6 — Additional file 6: Figure S3. Kaplan-Meier survival curves for the cancer-specific survival (CSS) of the MM patients according to CD138+ miR-25 levels. p values calculated by log-rank test. [file 12967_2023_4034_MOESM6_ESM.tif]

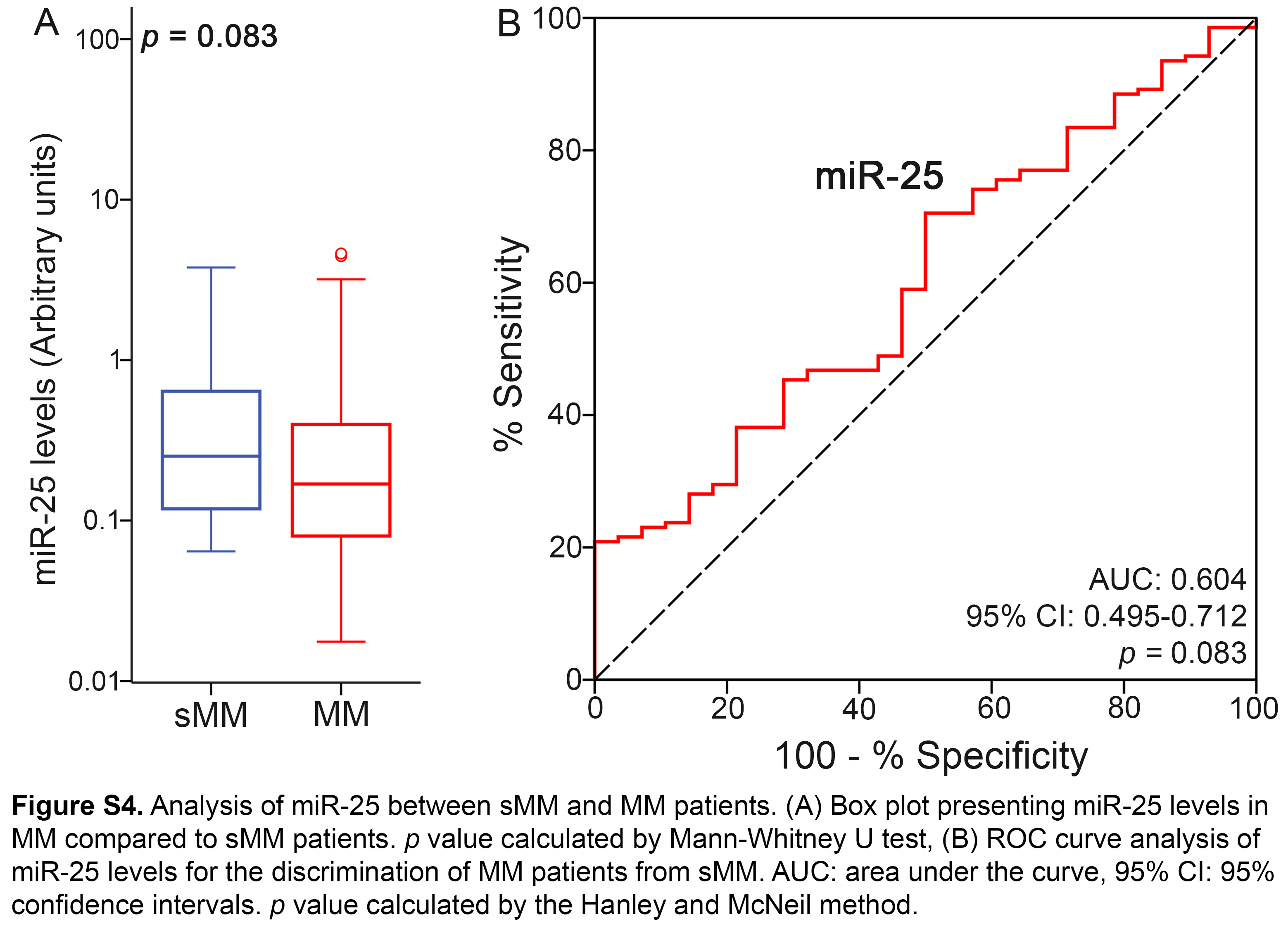

Supplement: Supplementary file 8 — Additional file 8: Figure S4. Analysis of miR-25 between sMM and MM patients. (A) Box plot presenting miR-25 levels in MM compared to sMM patients. p value calculated by Mann-Whitney U test, (B) ROC curve analysis of miR-25 levels for the discrimination of MM patients from sMM. AUC: area under the curve, 95% CI: 95% confidence intervals. p value calculated by the Hanley and McNeil method. [file 12967_2023_4034_MOESM8_ESM.tif]

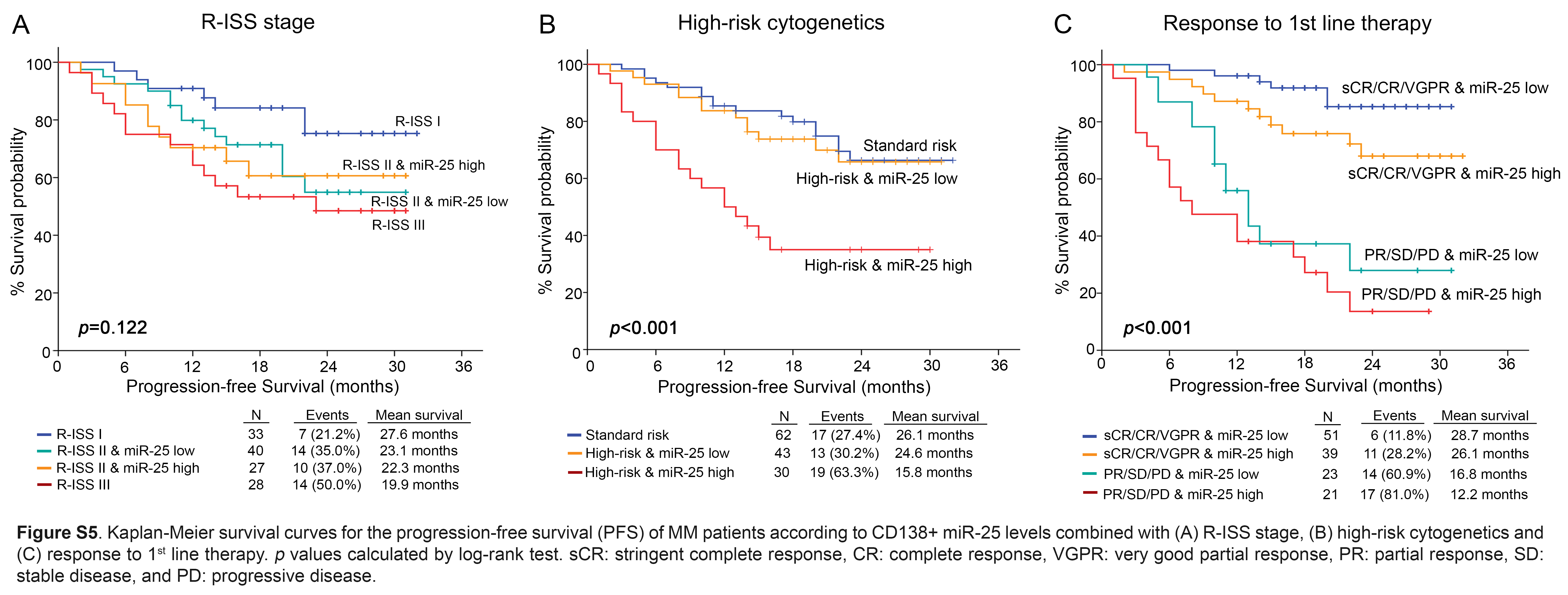

Supplement: Supplementary file 9 — Additional file 9: Figure S5. Kaplan-Meier survival curves for the progression-free survival (PFS) of MM patients according to CD138+ miR-25 levels combined with (A) R-ISS stage, (B) high-risk cytogenetics and (C) response to 1st line therapy. p values calculated by log-rank test. sCR: stringent complete response, CR: complete response, VGPR: very good partial response, PR: partial response, SD: stable disease, and PD: progressive disease. [file 12967_2023_4034_MOESM9_ESM.tif]

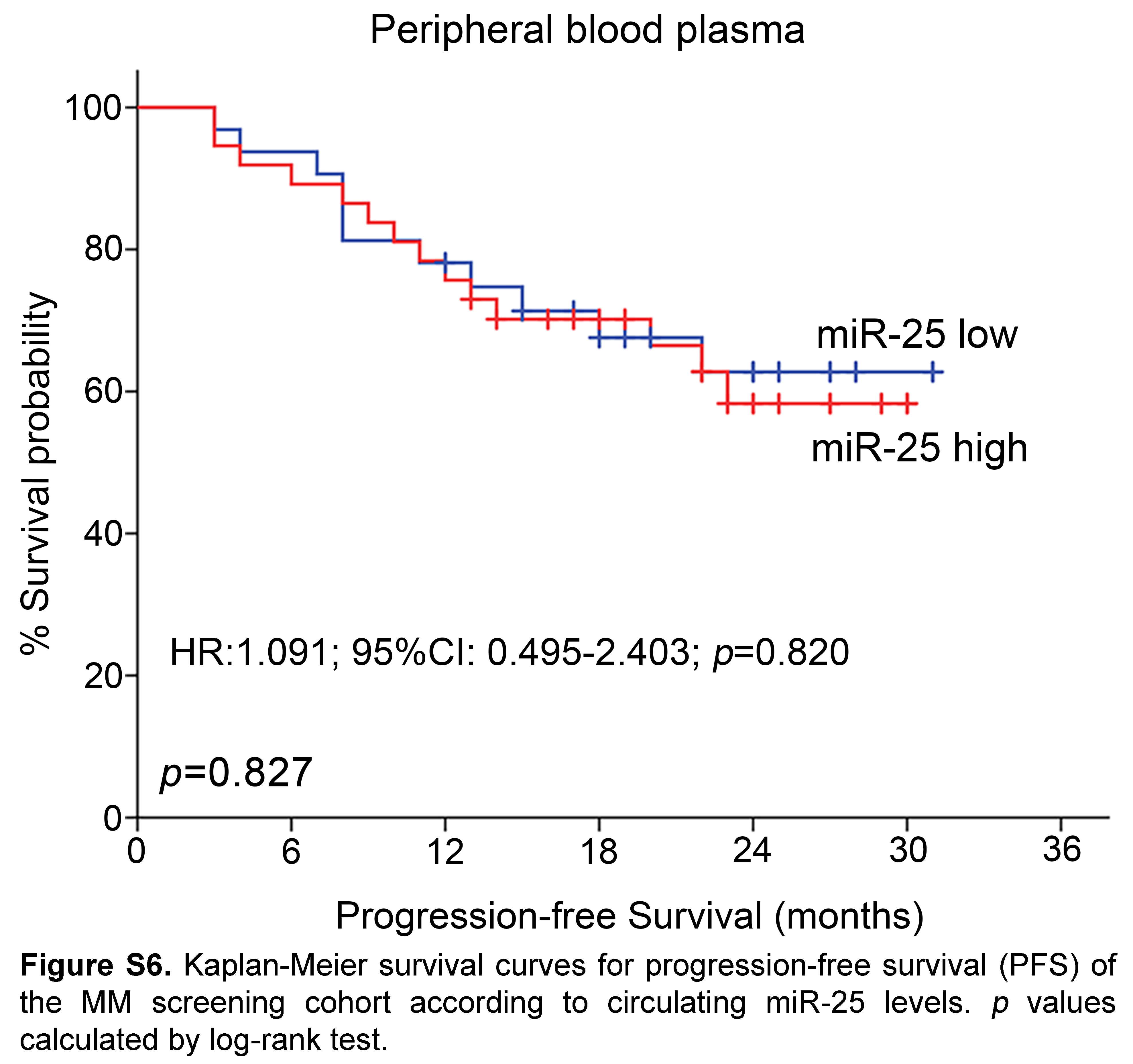

Supplement: Supplementary file 11 — Additional file 11: Figure S6. Kaplan-Meier survival curves for progression-free survival (PFS) of the MM screening cohort according to circulating miR-25 levels. p values calculated by log-rank test. [file 12967_2023_4034_MOESM11_ESM.tif]
