# Supplementary material for: miRNA-seq identification and clinical validation of CD138+ and circulating miR-25 in treatment response of multiple myeloma
Source: J Transl Med. 2023 Apr 6;21:245. doi: 10.1186/s12967-023-04034-5 (PMC10080848; doi:10.1186/s12967-023-04034-5)
Supplement: Supplementary file 7 — Additional file 7: Table S4. Cox regression analysis for the prediction of MM patients’ risk for death (OS) and progression (PFS) based on CD138+ miR-25 levels. [file 12967_2023_4034_MOESM7_ESM.docx]

**Table S4.** Cox regression analysis for thsse prediction of MM patients’ risk for death (OS) and progression (PFS) based on CD138+ miR-25 levels

|  | ***Univariate analysis*** | | | | | | | | | |
| --- | --- | --- | --- | --- | --- | --- | --- | --- | --- | --- |
|  | **Overall survival (OS)** | | | | | **Progression-free survival (PFS)** | | | | |
| **Covariant** | **HR^a^** | **95% CI^b^** | ***p*-value^c^** | **Bootstrap**  **BCa 95% CI^d^** | **Bootstrap**  ***p*-value^c^** | **HR^a^** | **95% CI^b^** | ***p*-value^c^** | **Bootstrap**  **BCa 95% CI^d^** | **Bootstrap**  ***p*-value^c^** |
| **CD138+ miR-25**  Low expression  High expression | 1.00  3.149 | 1.442-6.877 | 0.004 | 1.479-9.164 | 0.001 | 1.00  1.825 | 1.036-3.214 | 0.037 | 1.044-3.301 | 0.029 |
| **R-ISS Stage**  R-ISS I / II  R-ISS III | 1.00  2.415 | 1.130-5.158 | 0.023 | 0.991-5.554 | 0.022 | 1.00  1.776 | 0.944-3.340 | 0.075 | 0.828-3.289 | 0.079 |
| **High risk Cytogenetics**  No  Yes | 1.00  2.705 | 1.203-6.082 | 0.016 | 1.237-8.450 | 0.008 | 1.00  1.882 | 1.044-3.392 | 0.035 | 1.082-3.506 | 0.028 |
| **LDH**  ≤ 220 U/L  ≥ 220 U/L | 1.00  2.289 | 1.102-4.754 | 0.026 | 1.106-4.732 | 0.025 | 1.00  1.949 | 1.081-3.513 | 0.026 | 1.055-3.468 | 0.018 |
| **B2M**  ≤ 5.5 mg/L  ≥ 5.5 mg/L | 1.00  3.175 | 1.476-6.829 | 0.003 | 1.510-8.806 | 0.002 | 1.00  2.836 | 1.590-5.061 | <0.001 | 1.607-5.020 | 0.001 |
| **Creatinine**  ≤ 2 mg/dL  ≥ 2 mg/dL | 1.00  1.858 | 0.826-4.175 | 0.134 | 0.812-3.574 | 0.126 | 1.00  1.460 | 0.746-2.858 | 0.269 | 0.701-2.749 | 0.271 |
| **HDM/ASCT**  Yes  No | 1.00  2.459 | 0.856-7.066 | 0.095 | 0.906-30.50 | 0.060 | 1.00  2.952 | 1.255-6.945 | 0.013 | 1.288-12.64 | 0.018 |
| **Response to 1st line**  sCR, CR, VGPR  PR, SD, PD | 1.00  3.124 | 1.461-6.681 | 0.003 | 1.434-7.084 | 0.001 | 1.00  6.385 | 3.470-11.75 | <0.001 | 3.780-12.99 | 0.001 |
| **Gender**  Female  Male | 1.00  1.334 | 0.635-2.805 | 0.447 | 0.558-3.342 | 0.461 | 1.00  0.814 | 0.464-1.428 | 0.473 | 0.468-1.468 | 0.469 |
| **Age** (continuous) | 1.040 | 1.003-1.078 | 0.032 | 1.004-1.081 | 0.022 | 1.024 | 0.997-1.052 | 0.086 | 0.995-1.057 | 0.102 |
|  | ***Multivariate analysis^e^*** | | | | | | | | | |
|  | **Overall survival (OS)** | | | | | **Progression-free survival (PFS)** | | | | |
| **Covariant** | **HR^a^** | **95% CI^b^** | ***p*-value^c^** | **Bootstrap**  **BCa 95% CI^d^** | **Bootstrap**  ***p*-value^c^** | **HR^a^** | **95% CI^b^** | ***p*-value^c^** | **Bootstrap**  **BCa 95% CI^d^** | **Bootstrap**  ***p*-value^c^** |
| **CD138+ miR-25**  Low expression  High expression | 1.00  4.614 | 1.755-12.13 | 0.002 | 1.271-47.91 | 0.001 | 1.00  2.669 | 1.293-5.509 | 0.008 | 0.861 -18.37 | 0.023 |
| **R-ISS Stage**  R-ISS I / II  R-ISS III | 1.00  0.356 | 0.083-1.521 | 0.163 | 0.039-2.866 | 0.313 | 1.00  0.430 | 0.144-1.281 | 0.130 | 0.115-0.980 | 0.225 |
| **High risk Cytogenetics**  No  Yes | 1.00  2.465 | 0.842-7.217 | 0.100 | 0.220- 119.4 | 0.228 | 1.00  1.688 | 0.798-3.571 | 0.171 | 0.520-13.44 | 0.316 |
| **LDH**  ≤ 220 U/L  ≥ 220 U/L | 1.00  3.073 | 0.998-9.461 | 0.050 | 0.622-25.59 | 0.122 | 1.00  2.213 | 0.935-5.240 | 0.071 | 0.569-11.50 | 0.101 |
| **B2M**  ≤ 5.5 mg/L  ≥ 5.5 mg/L | 1.00  5.115 | 1.313-19.92 | 0.019 | 0.616-53.16 | 0.038 | 1.00  5.369 | 2.075-13.89 | 0.001 | 0.999-52.13 | 0.003 |
| **Creatinine**  ≤ 2 mg/dL  ≥ 2 mg/dL | 1.00  0.482 | 0.149-1.554 | 0.222 | 0.053-3.825 | 0.312 | 1.00  0.401 | 0.154-1.044 | 0.061 | 0.094-1.376 | 0.148 |
| **HDM/ASCT**  Yes  No | 1.00  0.879 | 0.198-3.908 | 0.865 | 0.011- 7.97x10^5^ | 0.853 | 1.00  2.348 | 0.789-6.985 | 0.125 | 0.535- 5.26x10^4^ | 0.168 |
| **Response to 1st line**  sCR, CR, VGPR  PR, SD, PD | 1.00  1.583 | 0.648-3.871 | 0.313 | 0.423-12.95 | 0.383 | 1.00  4.950 | 2.416-10.14 | <0.001 | 1.772-42.08 | 0.001 |
| **Gender**  Female  Male | 1.00  1.385 | 0.536-3.581 | 0.502 | 0.314-9.079 | 0.528 | 1.00  0.742 | 0.371-1.485 | 0.399 | 0.282-2.549 | 0.466 |
| **Age** (Continuous) | 1.041 | 0.980-1.106 | 0.188 | 0.974-1.116 | 0.190 | 0.973 | 0.935-1.012 | 0.172 | 0.929-1.002 | 0.266 |

a: Hazard Ratio, b: 95% confidence interval of the estimated HR, c: Bootstrap *p*-value is based on 1000 bootstrap samples d: Bootstrap bias-corrected and accelerated 95% CI of the estimated HR based on 1000 bootstrap samples, e: Multivariate analysis adjusted for CD138+ miR-25 levels, R-ISS, high-risk cytogenetics, B2M / LDH / creatinine levels, gender, age, and response to 1st line therapy.
