# Supplementary material for: miRNA-seq identification and clinical validation of CD138+ and circulating miR-25 in treatment response of multiple myeloma
Source: J Transl Med. 2023 Apr 6;21:245. doi: 10.1186/s12967-023-04034-5 (PMC10080848; doi:10.1186/s12967-023-04034-5)
Supplement: Supplementary file 10 — Additional file 10: Table S5. Cox regression analysis for the prediction of MM patients’ risk for death (OS) based on circulating miR-25 levels. [file 12967_2023_4034_MOESM10_ESM.docx]

**Table S5.** Cox regression analysis for the prediction of MM patients’ risk for death (OS) based on circulating miR-25 levels

|  | ***Univariate analysis*** | | | | | ***Multivariate analysis*** | | | | |
| --- | --- | --- | --- | --- | --- | --- | --- | --- | --- | --- |
|  | **Overall survival (OS)** | | | | | **Overall survival (OS)** | | | | |
| **Covariant** | **HR^a^** | **95% CI^b^** | ***p*-value^c^** | **Bootstrap**  **BCa 95% CI^d^** | **Bootstrap**  ***p*-value^c^** | **HR^a^** | **95% CI^b^** | ***p*-value^c^** | **Bootstrap**  **BCa 95% CI^d^** | **Bootstrap**  ***p*-value^c^** |
| **Circulating miR-25**  Low levels  High levels | 1.00  5.435 | 1.203-24.56 | 0.028 | 1.260-97.19 | 0.021 | 1.00  4.096 | 0.787-21.32 | 0.094 | 9.7x10^-30^- 4x10^172^ | 0.080 |
| **R-ISS Stage**  R-ISS I / II  R-ISS III | 1.00  1.867 | 0.545-6.390 | 0.320 | 0.366-6.750 | 0.316 | 1.00  1.586 | 0.115-21.80 | 0.730 | 1.8x10^-29^- 2x10^40^ | 0.478 |
| **High risk Cytogenetics**  No  Yes | 1.00  0.955 | 0.320-2.845 | 0.933 | 0.301-3.749 | 0.942 | 1.00  0.760 | 0.166-3.470 | 0.723 | 3x10^-8^- 5.914x10^4^ | 0.725 |
| **LDH**  ≤ 220 U/L  ≥ 220 U/L | 1.00  1.445 | 0.443-4.713 | 0.542 | 0.275-3.876 | 0.530 | 1.00  0.640 | 0.117-3.497 | 0.607 | 7.9x10^-7^-44.89 | 0.567 |
| **B2M**  ≤ 5.5 mg/L  ≥ 5.5 mg/L | 1.00  2.038 | 0.656-6.325 | 0.218 | 0.542-6.771 | 0.202 | 1.00  1.073 | 0.108-10.63 | 0.952 | 7x10^-32^-8.99x10^129^ | 0.883 |
| **Creatinine**  ≤ 2 mg/dL  ≥ 2 mg/dL | 1.00  0.947 | 0.210-4.279 | 0.944 | 0.038-3.093 | 0.948 | 1.00  0.767 | 0.072-8.150 | 0.826 | 1.7x10^-6^-33.93 | 0.706 |
| **HDM/ASCT**  Yes  No | 1.00  3.611 | 0.800-16.30 | 0.095 | 0.803-59.04 | 0.071 | 1.00  3.447 | 0.397-29.97 | 0.292 | 7.8x10^-17^-7.1x10^35^ | 0.297 |
| **Response to 1st line**  sCR, CR, VGPR  PR, SD, PD | 1.00  3.184 | 1.010-10.04 | 0.048 | 0.778-23.52 | 0.029 | 1.00  2.111 | 0.395-11.28 | 0.382 | 7x10^-120^-4x10^132^ | 0.507 |
| **Gender**  Female  Male | 1.00  2.577 | 0.709-9.370 | 0.151 | 0.739-49.22 | 0.103 | 1.00  8.713 | 0.999-76.01 | 0.050 |  | 0.024 |
| **Age** (Continuous) | 1.034 | 0.983-1.088 | 0.198 | 0.988-1.094 | 0.148 | 0.974 | 0.892-1.065 | 0.566 | 0.583-1.096 | 0.599 |

a: Hazard Ratio, b: 95% confidence interval of the estimated HR c: Bootstrap *p*-value is based on 1000 bootstrap samples, d: Bootstrap bias-corrected and accelerated 95% CI of the estimated HR based on 1000 bootstrap samples, e: Multivariate analysis adjusted for circulating miR-25 levels, R-ISS, high-risk cytogenetics, B2M / LDH / creatinine levels, gender, age, and response to 1st line therapy
